# Stability and error analysis for a diffuse interface approach to an advection–diffusion equation on a moving surface

**DOI:** 10.1007/s00211-018-0946-6

**Published:** 2018-01-25

**Authors:** Klaus Deckelnick, Vanessa Styles

**Affiliations:** 1 Fakultät für Mathematik, Otto-von-Guericke-Universität Magdeburg, 39106 Magdeburg, Germany; 2 Department of Mathematics, University of Sussex, Brighton, BN1 9QH UK

**Keywords:** 65M60, 65M15, 58J32

## Abstract

In this paper we analyze a fully discrete numerical scheme for solving a parabolic PDE on a moving surface. The method is based on a diffuse interface approach that involves a level set description of the moving surface. Under suitable conditions on the spatial grid size, the time step and the interface width we obtain stability and error bounds with respect to natural norms. Furthermore, we present test calculations that confirm our analysis.

## Introduction

Let $\{ \varGamma (t) \}_{t \in [0,T]}$ be a family of closed hypersurfaces in ${\mathbb {R}}^{n+1} (n=1,2)$ evolving in time. In this paper we consider a finite element approach for solving the parabolic surface PDE equation

1 $$\begin{aligned} \partial _t^{\bullet } u + u \nabla _{\varGamma } \cdot {\varvec{v}}- \varDelta _{\varGamma } u= & {} f \qquad \text{ on } S_T \end{aligned}$$

2 $$\begin{aligned} u(\cdot ,0)= & {} u_0 \quad \; \text{ on } \varGamma (0), \end{aligned}$$

which models advection and diffusion of a surface quantity *u* with $u(\cdot ,t):\varGamma (t) \rightarrow {\mathbb {R}}$. Here, $S_T= \bigcup _{t \in (0,T)} \bigl ( \varGamma (t) \times \lbrace t \rbrace \bigr )$ and ${\varvec{v}}: \overline{S_T} \rightarrow {\mathbb {R}}^{n+1}$ denotes a given velocity field. Furthermore, $\nabla _{\varGamma }$ is the tangential gradient, $\varDelta _{\varGamma } = \nabla _{\varGamma } \cdot \nabla _{\varGamma }$ the Laplace Beltrami operator and $\partial _t^{\bullet }=\partial _t + {\varvec{v}}\cdot \nabla$ denotes the material derivative.

Parabolic surface PDEs of the form (1) have applications in fluid dynamics and materials science, such as the transport and diffusion of surfactants on a fluid/fluid interface, [25] or diffusion-induced grain boundary motion, [5]. In these as in several other applications the velocity ${\varvec{v}}$ is not given but determined through an additional equation so that (1) becomes a subproblem of a more complicated system in which the variable *u* is coupled to other variables. The analysis and the numerical solution of such systems then naturally requires the development of corresponding methods for (1). We refer to [13] for a comprehensive overview of finite element methods for solving PDEs on stationary and evolving surfaces.

Concerning the numerical methods that have been proposed for (1) one may distinguish between Lagrangian and Eulerian type schemes. The first approach has been pursued by Dziuk and Elliott within their evolving surface finite element method, [8], which uses polyhedral approximations of the evolving hypersurfaces $\varGamma (t)$. While [8] contains an error analysis in the spatially discrete case, the fully discrete case is investigated in [11, 14] and [19]. Optimal $L^2$-error bounds are obtained in [12] and a corresponding finite volume approach is proposed and analyzed in [18]. Since the mesh for the discretization of (1) is fitted to the hypersurface $\varGamma (t)$, a coupling to a bulk equation is not straightforward. This difficulty is not present in Eulerian type schemes, in which $\varGamma (t)$ is typically described via a level set function defined in an open neighbourhood of $\varGamma (t)$. In order to discretize the surface PDE in this setting it has been proposed in [1, 3] and [27] to extend the surface quantity *u* to a band around $\varGamma (t)$ and to solve a suitable (weakly) parabolic PDE in that bulk region using a finite difference method. In [9] and [10], the same idea is used in a finite element context for which the underlying variational formulation is derived with the help of a transport identity. An Eulerian finite element approach that doesn’t use an extended PDE is proposed and analyzed in [20] and [21]. The method is based on a weak formulation on the space-time manifold and the finite element space is obtained by taking traces of the corresponding bulk finite elements. The approximation of $\varGamma (t)$ on which these spaces are defined usually arises from a suitable interpolation of the given level set function describing $\varGamma (t)$. The resulting discrete hypersurface will in general cut arbitrarily through the background mesh and its location forms one of the main difficulties in implementing the scheme. A different approach of generating the discrete hypersurfaces is pursued in [17], where a discretization of (4) below is combined with the cut finite element technique. Finally, Section 5 in [7] proposes a hybrid method that employs the above–mentioned idea of trace finite elements together with a narrow band technique for the elliptic part of the PDE.

In this paper we are concerned with the diffuse interface approach for solving (1), which was introduced in [22] for a stationary surface and in [16, 23] and [26] for evolving surfaces. As in some of the methods described above, the surface quantity *u* is extended to a bulk quantity satisfying a suitable parabolic PDE in a neighbourhood of $\varGamma (t)$ and the bulk equation is then localized to a thin layer of thickness $\epsilon$ with the help of a phase field function (see [15] for a corresponding convergence analysis). Since we are interested in using finite elements, the localized PDE needs to be written in a suitable variational form. Following [16] this is achieved with the help of a transport identity and results in a discretization by linear finite elements in space and a backward Euler scheme in time. The detailed derivation along with an existence result for the discrete solution will be given in Sect. 3. As the main new contribution of our paper we shall derive conditions relating the interface width $\epsilon$, the spatial grid size *h* and the time step $\tau$ which allow for a rigorous stability and error analysis. More precisely, we shall prove that the numerical solution is bounded uniformly in $L^{\infty }(L^2)$ and $L^2(H^1)$ over the diffuse interface (see Theorem 1 in Sect. 4) and that it converges with respect to these norms both over the diffuse interface and on the sharp interface with an order $O(\epsilon )$ provided that

$$\begin{aligned} h \le c_1 \epsilon , \; \tau \le c_2 \epsilon ^2, \end{aligned}$$

see Theorem 2 and Corollary 1 in Sect. 5 respectively. In Sect. 6 we report on results of numerical tests both for $n=1$ and $n=2$.

An advantage of our approach is that in the implementation the evolution of the hypersurfaces is easily incorporated by evaluating the phase field function. We shall employ a function with compact support, namely $\rho (x,t):= g(\frac{\phi (x,t)}{\epsilon })$, where $\varGamma (t)$ is the zero level set of $\phi (\cdot ,t)$ and

$$\begin{aligned} g(r)= \left\{ \begin{array}{ll} \cos ^2(r), &{} | r | \le \frac{\pi }{2}, \\ 0, &{} |r| > \frac{\pi }{2}. \end{array} \right. \end{aligned}$$

In view of the evolution of the hypersurfaces the numerical scheme then naturally contains terms in which $\rho$ is evaluated at different times. One of the main challenges in the analysis is to handle the corresponding differences, for which one has to bound integrals that are multiplied by a negative power of $\epsilon$ (arising from derivatives of $\rho$) as well as integrals that are not weighted with $\rho$. We shall deal with these difficulties by introducing an additional stabilization term with extended support that is also used for proving the well–posedness of the scheme.

Let us finally remark that a phase field approach involving a phase field function with noncompact support and finite elements has been proposed in [4] for an elliptic surface PDE. Theorem 7 in [4] provides an error estimate in terms of an approximation error and an error due to the phase field representation. The latter decays at a rate $O(\epsilon ^p)$ for some $p<1$, while a coupling between $\epsilon$ and the grid size *h* is not discussed.

## Preliminaries

### Surface representation and surface derivatives

For each $t \in [0,T]$ let $\varGamma (t) \subset {\mathbb {R}}^{n+1} \, (n=1,2)$ be a connected, compact and orientable hypersurface without boundary. We suppose that ${\varvec{v}}: \overline{S_T} \rightarrow {\mathbb {R}}^{n+1}$ is a prescribed velocity field of the form

3 $$\begin{aligned} {\varvec{v}}= V \nu + {\varvec{v_\tau }}, \quad \text{ with } ({\varvec{v_\tau }}, \nu )=0. \end{aligned}$$

Here, $\nu$ is a unit normal and *V* the corresponding normal velocity of $\varGamma (t)$ and $(\cdot , \cdot )$ denotes the Euclidian scalar product in ${\mathbb {R}}^{n+1}$. Note that the normal part $V\nu$ is responsible for the geometric motion of $\varGamma (t)$, while the tangential part ${\varvec{v_\tau }}$ is associated with the transport of material along the surface. We assume that there exists a smooth map $\varPsi :\varGamma (0) \times [0,T] \rightarrow {\mathbb {R}}^{n+1}$ such that $\varPsi (\cdot ,t)$ is a diffeomorphism from $\varGamma (0)$ onto $\varGamma (t)$ for every $t \in [0,T]$ satisfying

4 $$\begin{aligned} \frac{\partial \varPsi }{\partial t}(P,t)= & {} {\varvec{v}}(\varPsi (P,t),t), \quad P \in \varGamma (0), t \in (0,T]; \end{aligned}$$

5 $$\begin{aligned} \varPsi (P,0)= & {} P, \qquad \qquad \quad \; \, P \in \varGamma (0). \end{aligned}$$

Let us next introduce the differential operators which are required to formulate our PDE. To begin, for fixed *t* and a function $\eta : \varGamma (t) \rightarrow {\mathbb {R}}$ we denote by $\nabla _{\varGamma } \eta =(\underline{D}_1 \eta ,\ldots , \underline{D}_{n+1}\eta )$ its tangential gradient. If $\bar{\eta }$ is an extension of $\eta$ to an open neighbourhood of $\varGamma (t)$ then

6 $$\begin{aligned} \nabla _{\varGamma } \eta (x) = \bigl ( I- \nu (x,t) \otimes \nu (x,t) \bigr ) \nabla \bar{\eta }(x), \quad x \in \varGamma (t). \end{aligned}$$

Furthermore, $\varDelta _{\varGamma } \eta = \nabla _{\varGamma } \cdot \nabla _{\varGamma } \eta = \sum _{i=1}^{n+1} \underline{D}_i \underline{D}_i \eta$ denotes the Laplace-Beltrami operator.

Next, for a smooth function $\eta$ on $S_T$ we define the material derivative of $\eta$ at $(x,t)=(\varPsi (P,t),t)$ by $\partial _t^{\bullet }\eta (x,t):= \frac{d}{dt} [ \eta (\varPsi (P,t),t)]$. If $\bar{\eta }$ is an extension of $\eta$ to an open space-time neighbourhood, then

$$\begin{aligned} \partial _t^{\bullet } \eta (x,t) = \bar{\eta }_t(x,t) + ({\varvec{v}}(x,t),\nabla \bar{\eta }(x,t)), \quad (x,t) \in S_T. \end{aligned}$$

Our numerical approach will be based on an implicit representation of $\varGamma (t)$, so that we suppose in what follows that there exists a smooth function $\phi : \varOmega \times [0,T] \rightarrow {\mathbb {R}}$ such that for $0 \le t \le T$

7 $$\begin{aligned} \displaystyle \varGamma (t) = \lbrace x \in \varOmega \, | \, \phi (x,t) = 0 \rbrace \quad \text{ and } \quad \nabla \phi (x,t) \ne 0, \, x \in \varGamma (t). \end{aligned}$$

Here, $\varOmega \subset {\mathbb {R}}^{n+1}$ is a bounded domain with $\varGamma (t) \subset \varOmega$ for all $t \in [0,T]$. For later use we introduce for $t \in [0,T], \, r>0$ the sets

$$\begin{aligned} U_r(t):= \lbrace x \in \varOmega \, | \, | \phi (x,t) | <r \rbrace \quad \text{ and } \quad {\mathcal {U}}_{r,T}:= \bigcup _{t \in [0,T]} \bigl ( U_r(t) \times \lbrace t \rbrace \bigr ). \end{aligned}$$

In view of (7) there exist $\delta _0>0, 0< c_0 \le c_1, \, c_2>0$ such that $\overline{U_{\delta _0}(t)} \subset \varOmega , 0 \le t \le T$ and

8 $$\begin{aligned} \displaystyle c_0 \le | \nabla \phi (x,t) | \le c_1, \; | D^2 \phi (x,t) |, \, | \phi _t(x,t) |, \, | \phi _{tt}(x,t) | \le c_2, \quad (x,t) \in {\mathcal {U}}_{\delta _0,T}. \end{aligned}$$

### Extension

Our next aim is to extend functions defined on $S_T$ to a space-time neighbourhood. A common approach which is well suited to a description of $\varGamma (t)$ via the signed distance function consists in extending constantly in the normal direction. In what follows we shall introduce a suitable generalization to the case (7). Consider for $P \in \varGamma (0)$ and $t \in [0,T]$ the parameter-dependent system of ODEs

9 $$\begin{aligned} \gamma _{P,t}'(s) = \frac{\nabla \phi (\gamma _{P,t}(s),t)}{| \nabla \phi (\gamma _{P,t}(s),t) |^2}, \quad \gamma _{P,t}(0)= \varPsi (P,t). \end{aligned}$$

Using a compactness argument it can be shown that there exists $0< \delta < \delta _0$ so that the solution $\gamma _{P,t}$ of (9) exists uniquely on $(-\delta ,\delta )$ uniformly in $P \in \varGamma (0), t \in [0,T]$. Thus we can define the smooth mapping $F_t:\varGamma (0) \times (-\delta ,\delta ) \rightarrow {\mathbb {R}}^{n+1}$ by

10 $$\begin{aligned} F_t(P,s):=\gamma _{P,t}(s), \quad P \in \varGamma (0), |s| < \delta . \end{aligned}$$

In view of the chain rule and (9) we immediately see that $\frac{d}{ds} \phi (\gamma _{P,t}(s),t) = 1$, which implies that $\phi (\gamma _{P,t}(s),t)=s, |s| < \delta$ since $\gamma _{P,t}(0)=\varPsi (P,t) \in \varGamma (t)$. In particular, $x=F_t(P,s)$ yields that $| \phi (x,t) | < \delta$ and it is not difficult to verify that $F_t$ is a diffeomorphism of $\varGamma (0) \times (-\delta ,\delta )$ onto $U_{\delta }(t)$ for $t \in [0,T]$, whose inverse has the form

11 $$\begin{aligned} F_t^{-1}(x)= (p(x,t),\phi (x,t)), \quad x \in U_{\delta }(t). \end{aligned}$$

Here, $p: {\mathcal {U}}_{\delta ,T} \rightarrow {\mathbb {R}}^{n+1}$ satisfies $p(x,t) \in \varGamma (0), x \in U_{\delta }(t)$. Furthermore, since $\phi (F_t(P,s),t)=s$ we deduce from (11) that

12 $$\begin{aligned} \displaystyle p(x,t) = P, \quad \text{ if } x= F_t(P,s) \in U_{\delta }(t). \end{aligned}$$

The function $\tilde{p}: {\mathcal {U}}_{\delta ,T} \rightarrow {\mathbb {R}}^{n+1}, \, \tilde{p}(x,t):= \varPsi (p(x,t),t)$ then is smooth and satisfies $\tilde{p}(x,t) \in \varGamma (t), 0 \le t \le T$. In addition we claim that

13 $$\begin{aligned} \displaystyle \tilde{p}(x,t) = x, \quad x \in \varGamma (t). \end{aligned}$$

To see this, let $x \in \varGamma (t)$, say $x=\varPsi (P,t)=\gamma _{P,t}(0)=F_t(P,0)$ for some $P \in \varGamma (0)$. Using (12) with $s=0$ we deduce that

$$\begin{aligned} \tilde{p}(x,t)= \varPsi (p(x,t),t) = \varPsi (P,t) = x, \end{aligned}$$

proving (13). Let us next use $\tilde{p}$ in order to extend a function $z: \overline{S_T} \rightarrow {\mathbb {R}}$ to ${\mathcal {U}}_{\delta ,T}$ by setting

14 $$\begin{aligned} \displaystyle z^e(x,t):= z(\tilde{p}(x,t),t), \quad (x,t) \in {\mathcal {U}}_{\delta ,T}. \end{aligned}$$

Clearly, $z^e(\cdot ,t)=z(\cdot ,t)$ on $\varGamma (t)$ by (13). Moreover, (12) implies for $P \in \varGamma (0), |s| < \delta$

$$\begin{aligned} z^e(F_t(P,s),t) = z \bigl ( \tilde{p}(F_t(P,s),t),t \bigr )= z \bigl (\varPsi (p(F_t(P,s),t),t),t \bigr ) = z(\varPsi (P,t),t), \end{aligned}$$

from which we obtain by differentiating with respect to *s* and using (9), (10) that

15 $$\begin{aligned} \displaystyle \bigl ( \nabla z^e(x,t),\nabla \phi (x,t) \bigr ) = 0, \quad (x,t) \in {\mathcal {U}}_{\delta ,T}. \end{aligned}$$

#### Lemma 1

Let $z^e$ be defined by (14). Then we have for $t \in [0,T], \, 0< r < \delta$ and $| \alpha | =k \in \lbrace 0,1,2 \rbrace$:

16 $$\begin{aligned} \Vert D_x^{\alpha } z^e(\cdot ,t) \Vert _{L^2(U_r(t))}\le & {} C \sqrt{r} \Vert z(\cdot ,t) \Vert _{H^k(\varGamma (t))}; \end{aligned}$$

17 $$\begin{aligned} \Vert D_x^{\alpha } z^e_t(\cdot ,t) \Vert _{L^2(U_r(t))}\le & {} C \sqrt{r} \bigl ( \Vert \partial ^{\bullet }_t z (\cdot ,t) \Vert _{H^k(\varGamma (t))} + \Vert z(\cdot ,t) \Vert _{H^{k+1}(\varGamma (t))} \bigr ). \end{aligned}$$

#### Proof

Let us recall that $F_t$ is a diffeomorphism from $\varGamma (0) \times (-r,r)$ onto $U_r(t)$ while $\varPsi (\cdot ,t)$ is a diffeomorphism from $\varGamma (0)$ onto $\varGamma (t)$. We deduce from (12) and the definition of $\tilde{p}$ that $\tilde{p}(F_t(P,s),t)=\varPsi (P,t), P \in \varGamma (0), | s| < r$ so that we obtain with the help of the transformation rule

18 $$\begin{aligned} \displaystyle \int _{U_r(t)} | z^e(x,t) |^2 dx= & {} \int _{U_r(t)} | z(\tilde{p}(x,t),t) |^2 dx \le c \int _{-r}^r \int _{\varGamma (0)} | z(\varPsi (P,t),t) |^2 do_P ds \nonumber \\\le & {} c r \int _{\varGamma (t)} | z(Q,t) |^2 do_Q \end{aligned}$$

which is (16) for $k=0$. Next, differentiating the identity $\phi (\tilde{p}(x,t),t)=0$ with respect to $x_i$ we infer that $(\nabla \phi (\tilde{p}(x,t),t),\tilde{p}_{x_i}(x,t))=0,i=1,\ldots ,n+1$. Hence we obtain from (14) and (6) that

19 $$\begin{aligned} z^e_{x_i}(x,t)= & {} \sum _{k=1}^{n+1} z^e_{x_k}(\tilde{p}(x,t),t) \tilde{p}_{k,x_i}(x,t) = \sum _{k=1}^{n+1} \underline{D}_k z(\tilde{p}(x,t),t) \tilde{p}_{k,x_i}(x,t), \end{aligned}$$

20 $$\begin{aligned} z^e_{x_i x_j}(x,t)= & {} \sum _{k,l=1}^{n+1} \underline{D}_l \underline{D}_k z(\tilde{p}(x,t),t) \tilde{p}_{k,x_i}(x,t) \tilde{p}_{l,x_j}(x,t)\nonumber \\&+ \sum _{k=1}^{n+1} \underline{D}_k z(\tilde{p}(x,t),t) \tilde{p}_{k,x_i x_j}(x,t). \end{aligned}$$

Similarly, $(\nabla \phi (\tilde{p}(x,t),t),\tilde{p}_t(x,t)) = - \phi _t(\tilde{p}(x,t),t)=(\nabla \phi (\tilde{p}(x,t),t), {\varvec{v}}(\tilde{p}(x,t),t))$ by (24) below, so that

21 $$\begin{aligned} z^e_t(x,t)= & {} z^e_t(\tilde{p}(x,t),t) + (\nabla z^e(\tilde{p}(x,t),t), \tilde{p}_t(x,t)) \nonumber \\= & {} \partial ^{\bullet }_t z(\tilde{p}(x,t),t) + \sum _{k=1}^{n+1} \underline{D}_k z(\tilde{p}(x,t),t) \bigl ( \tilde{p}_{k,t}(x,t) - {\varvec{v}}_k(\tilde{p}(x,t),t) \bigr ). \qquad \end{aligned}$$

Combining (19), (20) with the argument in (18) we obtain (16). The estimate (17) follows in a similar way if one starts from (21). $\square$

Let us next extend the surface differential operators $\nabla _{\varGamma }$ and $\partial ^{\bullet }_t$. By reversing the orientation of $\varGamma (t)$ if necessary we may assume that the functions $\nu : {\mathcal {U}}_{\delta ,T} \rightarrow {\mathbb {R}}^{n+1}$, $V: {\mathcal {U}}_{\delta ,T} \rightarrow {\mathbb {R}}$ defined by

$$\begin{aligned} \nu (x,t):= \frac{ \nabla \phi (x,t)}{| \nabla \phi (x,t) |}, \quad V(x,t):= - \frac{\phi _t(x,t)}{| \nabla \phi (x,t) |}, \quad (x,t) \in {\mathcal {U}}_{\delta ,T} \end{aligned}$$

are extensions of the unit normal and the normal velocity respectively. In particular, we define for a function $\eta \in C^1(U_{\delta }(t))$ its Eulerian tangential gradient by

22 $$\begin{aligned} \displaystyle \nabla _{\phi } \eta (x):= \bigl ( I - \nu (x,t) \otimes \nu (x,t) \bigr ) \nabla \eta (x), \quad x \in U_{\delta }(t) \end{aligned}$$

and remark that $(\nabla _{\phi } \eta )_{| \varGamma (t)} = \nabla _{\varGamma } [\eta _{| \varGamma (t)}]$. Furthermore, it follows from Lemma 2 in [10] that for $\eta \in C^1_0(\varOmega )$ with $\text{ supp }\eta \subset U_{\delta }(t)$

23 $$\begin{aligned} \displaystyle \int _{\varOmega } \nabla _{\phi } \eta \, | \nabla \phi | = - \int _{\varOmega } \eta H \nu \, | \nabla \phi |, \quad \text{ where } H= - \nabla \cdot \nu . \end{aligned}$$

Note that $H_{| \varGamma (t)}$ is the mean curvature of $\varGamma (t)$.

Let us also extend the velocity field ${\varvec{v}}$ to ${\mathcal {U}}_{\delta ,T}$. We first extend its tangential part by setting

$$\begin{aligned} {\tilde{\mathbf{v}}_{\tau }}(x,t):= (I - \nu (x,t) \otimes \nu (x,t)) {\varvec{v_\tau }}^e(x,t), \quad (x,t) \in {\mathcal {U}}_{\delta ,T}. \end{aligned}$$

In view of (3) the function ${\varvec{v}}(x,t):= V(x,t) \nu (x,t) + {\tilde{\mathbf{v}}_{\tau }}(x,t)$ extends the given velocity field from $\overline{S_T}$ to ${\mathcal {U}}_{\delta ,T}$ and satisfies

24 $$\begin{aligned} \displaystyle \phi _t + ( {\varvec{v}}, \nabla \phi ) =0 \quad \text{ in } {\mathcal {U}}_{\delta ,T}. \end{aligned}$$

In particular, we can use the extended velocity ${\varvec{v}}$ to define the material derivative for a function $\eta$ on ${\mathcal {U}}_{\delta ,T}$ by setting

$$\begin{aligned} \partial _t^{\bullet } \eta (x,t):= \eta _t(x,t) + ({\varvec{v}}(x,t),\nabla \eta (x,t)), \quad (x,t) \in {\mathcal {U}}_{\delta ,T}. \end{aligned}$$

## Weak formulation and numerical scheme

### Phase field approach

Consider for $0< \epsilon < \frac{2 \delta }{\pi }$ the function

$$\begin{aligned} \rho (x,t):= g \left( \frac{\phi (x,t)}{\epsilon } \right) , \end{aligned}$$

where $g \in C^{1,1}({\mathbb {R}})$ is given by

$$\begin{aligned} g(r)= \left\{ \begin{array}{ll} \cos ^2(r), &{}\quad | r | \le \frac{\pi }{2}, \\ 0, &{}\quad |r| > \frac{\pi }{2}. \end{array} \right. \end{aligned}$$

Note that $\text{ supp }[\rho (\cdot ,t)] = \overline{U_{\frac{\epsilon \pi }{2}}(t)} \subset U_{\delta }(t)$. Furthermore, we obtain from the definition of $\nabla _{\phi }$ and (24)

25 $$\begin{aligned} \nabla _{\phi } \rho= & {} \frac{1}{\epsilon } g'\left( \frac{\phi }{\epsilon }\right) \nabla _{\phi } \phi =0, \end{aligned}$$

26 $$\begin{aligned} \partial _t^{\bullet } \rho= & {} \frac{1}{\epsilon } g'\left( \frac{\phi }{\epsilon }\right) \bigl ( \phi _t + ({\varvec{v}},\nabla \phi ) \bigr )=0. \end{aligned}$$

The phase field function $\rho$ allows us to approximate the integration over a surface $\varGamma (t)$ in terms of a volume integral over the diffuse interface. More precisely, for fixed $t \in [0,T]$, the coarea formula implies for $\eta \in L^1(\varOmega )$

$$\begin{aligned} \int _{\varOmega } \eta \, \rho (\cdot ,t) \, | \nabla \phi (\cdot ,t) | \, dx = \int _{- \frac{\epsilon \pi }{2}}^{ \frac{\epsilon \pi }{2}} g \left( \frac{s}{\epsilon } \right) \int _{\lbrace \phi (\cdot ,t)=s \rbrace } \eta \, d {\mathcal {H}}^n ds \approx \frac{\epsilon \pi }{2} \int _{\lbrace \phi (\cdot ,t)=0 \rbrace } \eta \, d {\mathcal {H}}^n \end{aligned}$$

for small $\epsilon >0$, so that we can view $\frac{2}{\epsilon \pi } \int _{\varOmega } \eta \, \rho (\cdot ,t) \, | \nabla \phi (\cdot ,t) | \, dx$ as an approximation of $\int _{\varGamma (t)} \eta \, d {\mathcal {H}}^n$. This formula explains the appearance of the weight $\rho (\cdot ,t) \, | \nabla \phi (\cdot ,t) |$ in subsequent volume integrals.

In what follows we shall make use of the following continuity properties of $\rho$.

#### Lemma 2

Let $s,t \in [0,T]$ with $| s-t | < \frac{\pi }{4 c_2} \epsilon$, $c_2$ as in (8). Then $\text{ supp }[ \rho (\cdot ,s)] \subset U_{\frac{3 \epsilon \pi }{4}}(t)$ and

27 $$\begin{aligned} | \rho (\cdot ,t) - \rho (\cdot ,s) |\le & {} C \frac{|t-s|}{\epsilon } \sqrt{\rho (\cdot ,t)} + C \frac{(t-s)^2}{\epsilon ^2} \chi _{U_{\frac{3\epsilon \pi }{4}}(t)} \quad \text{ in } \varOmega ; \end{aligned}$$

28 $$\begin{aligned} | \rho _t(\cdot ,t) - \rho _t(\cdot ,s) |\le & {} C \frac{|t-s|}{\varepsilon ^2} \chi _{U_{\frac{3\epsilon \pi }{4}}(t)} \quad \text{ in } \varOmega . \end{aligned}$$

#### Proof

Let $s, t \in [0,T]$ with $| s-t| < \frac{\pi }{4 c_2} \epsilon$ and $x \in \text{ supp }[ \rho (\cdot ,s)]= \overline{U_{\frac{\epsilon \pi }{2}}(s)}$. Using the mean value theorem and (8) we then have

$$\begin{aligned} | \phi (x, t)| \le | \phi (x,s)| + | \phi _t(x,\xi ) | \, | t - s| \le \frac{\epsilon \pi }{2}+ c_2 | t-s| < \frac{3 \epsilon \pi }{4}, \end{aligned}$$

i.e. $x \in U_{\frac{3 \epsilon \pi }{4}}(t)$. In order to prove (27) and (28) we first observe that it is enough to verify the estimates for $x \in U_{\frac{3\epsilon \pi }{4}}(t)$ in view of what we have just shown. There exists $\xi$ between *s* and *t* such that

29 $$\begin{aligned} | \rho (x,t) - \rho (x,s) |= & {} | \rho _t(x,\xi ) | \, | t-s| = \frac{1}{\epsilon } | \phi _t(x,\xi ) | \, \left| g'\left( \frac{\phi (x,\xi )}{\varepsilon }\right) \right| \, | t-s| \nonumber \\\le & {} \frac{c_2 |t-s|}{\epsilon } \left| g'\left( \frac{\phi (x,\xi )}{\varepsilon }\right) \right| \end{aligned}$$

by (8). Furthermore, since

$$\begin{aligned} g'(r)= \left\{ \begin{array}{ll} -2 \sin (r) \cos (r), &{}\quad | r | \le \frac{\pi }{2}, \\ 0, &{}\quad |r| > \frac{\pi }{2} \end{array} \right. \end{aligned}$$

we see immediately that

30 $$\begin{aligned} \displaystyle | g'(r) | \le 2 \sqrt{g(r)}, \quad | g'(r) - g'(\tilde{r})| \le 2 | r - \tilde{r}|, \quad r, \tilde{r} \in {\mathbb {R}}. \end{aligned}$$

As a result,

$$\begin{aligned} \left| g'\left( \frac{\phi (x,\xi )}{\varepsilon }\right) \right| \le \left| g'\left( \frac{\phi (x,t)}{\varepsilon }\right) \right| + \frac{2}{\epsilon } | \phi (x,\xi ) - \phi (x,t)| \le 2 \sqrt{\rho (x,t)} + \frac{2 c_2 |t-s| }{\epsilon }. \end{aligned}$$

Inserting this bound into (29) yields (27). Finally, using again (30) and (8) we obtain for $x \in U_{\frac{3\epsilon \pi }{4}}(t)$

$$\begin{aligned} | \rho _t(x,t) - \rho _t(x,s) |\le & {} \frac{1}{\epsilon } \left| g'\left( \frac{\phi (x,t)}{\epsilon } \right) - g'\left( \frac{\phi (x,s)}{\epsilon } \right) \right| \, | \phi _t(x,t) | \\&\quad + \frac{1}{\varepsilon } \left| g'\left( \frac{\phi (x,s)}{\epsilon } \right) \right| \, | \phi _t(x,t) - \phi _t(x,s)| \\\le & {} \frac{C}{\epsilon ^2} | \phi (x,t) - \phi (x,s) | + \frac{C}{\varepsilon } | \phi _t(x,t) - \phi _t(x,s) | \le \frac{C}{\varepsilon ^2} | t-s|. \end{aligned}$$

$$\square $$

#### Remark 1

Our analysis will work for other profile functions *g* than the one chosen above as long as they satisfy $g \in C^{1,1}({\mathbb {R}})$ and $g(r)>0$ if $|r| <R$, $g(r)=0$ if $|r| \ge R$ as well as $|g'(r)| \le C \sqrt{g(r)}$ for suitable $R,C>0$. Profile functions with noncompact support have been used in [4, 22] and [26]. However it is not obvious how to extend the analysis presented below to that setting.

### Discretization

Suppose that *u* is a smooth solution of (1). It is shown in Lemma 8 of the “Appendix” that its extension $u^e$ satisfies the strictly parabolic PDE

31 $$\begin{aligned} \partial _t^{\bullet } u^e + u^e \, \nabla _{\phi } \cdot {\varvec{v}}- \frac{1}{| \nabla \phi | } \nabla \cdot \bigl ( | \nabla \phi | \, \nabla u^e \bigr ) = f^e + \phi \, R \quad \text{ in } {\mathcal {U}}_{\delta ,T}, \end{aligned}$$

where

32 $$\begin{aligned} \displaystyle R(x,t)= & {} \sum _{k,l=1}^{n+1} b_{lk}(x,t) \underline{D}_l \underline{D}_k u(\tilde{p}(x,t),t) + \sum _{k=1}^{n+1} c_k(x,t) \underline{D}_k u(\tilde{p}(x,t),t) \nonumber \\&\quad +\,d(x,t) u(\tilde{p}(x,t),t) \end{aligned}$$

and $b_{lk},c_k,d$ are smooth functions depending on $\phi$ and ${\varvec{v}}$.

In order to associate with (31) a suitable variational formulation we adapt an idea from [16], which uses an Eulerian transport identity. More precisely, we infer with the help of Lemma 3 in [10], (26) and (31) that for every $\eta \in H^1(\varOmega )$

33 $$\begin{aligned} \frac{d}{dt}\int _\varOmega u^e\eta \, \rho \, | \nabla \phi |= & {} \int _\varOmega \bigl ( \partial _t^{\bullet }( u^e\eta \rho ) + u^e\eta \rho \, \nabla _{\phi } \cdot {\varvec{v}}\bigr ) | \nabla \phi |\nonumber \\= & {} \int _\varOmega \eta \bigl ( \partial _t^{\bullet } u^e + u^e \, \nabla _{\phi }\cdot {\varvec{v}}\bigr ) \rho \, | \nabla \phi |+ \int _\varOmega u^e \partial _t^{\bullet } \eta \, \rho \, | \nabla \phi |\nonumber \\= & {} \int _\varOmega \eta \, \nabla \cdot \bigl ( | \nabla \phi |\, \nabla u^e\bigr ) \rho + \int _\varOmega \eta \bigl ( f^e+\phi R \bigr ) \rho \, | \nabla \phi |\nonumber \\&+ \int _\varOmega u^e\partial _t^{\bullet } \eta \, \rho \, | \nabla \phi |=-\int _\varOmega (\nabla u^e, \nabla \eta ) \rho \, | \nabla \phi |+\int _\varOmega f^e\eta \, \rho \, | \nabla \phi |\nonumber \\&+\int _\varOmega u^e({\varvec{v}},\nabla \eta ) \rho \, | \nabla \phi |+\int _\varOmega \phi \, R \, \eta \, \rho \, | \nabla \phi |. \end{aligned}$$

Here, the last equality follows from integration by parts together with the fact that $(\nabla u^e, \nabla \rho )= \frac{1}{\epsilon } g'\left( \frac{\phi }{\epsilon } \right) (\nabla u^e,\nabla \phi )=0$ in view of (15).

Let us first discretize with respect to time and denote by $0=t_0< t_1< \cdots < t_M = T$ a partioning of [0, *T*] with time steps $\tau _m:=t_m - t_{m-1}$ and $\tau := \max _{m=1,\ldots ,M} \tau _m$. For a function $f=f(x,t)$ we shall write $f^m(x)=f(x,t_m)$. Integrating (33) with respect to $t \in (t_{m-1},t_m)$ we obtain for $\eta \in H^1(\varOmega )$

34 $$\begin{aligned}&\int _\varOmega u^{e,m} \eta \rho ^m|\nabla \phi ^{m}|-\int _\varOmega u^{e,m-1} \eta \rho ^{m-1}| \nabla \phi ^{m-1}|+\int _{t_{m-1}}^{t_m}\int _\varOmega (\nabla u^e, \nabla \eta ) \rho \, | \nabla \phi |\nonumber \\&\quad - \int _{t_{m-1}}^{t_m}\int _\varOmega u^e ({\varvec{v}},\nabla \eta ) \rho \, | \nabla \phi |= \int _{t_{m-1}}^{t_m}\int _\varOmega f^e\, \eta \, \rho \, | \nabla \phi |+ \int _{t_{m-1}}^{t_m}\int _\varOmega \phi \, R \, \eta \, \rho \, | \nabla \phi |.\nonumber \\ \end{aligned}$$

Under a suitable regularity assumption on *u* we have that $| \phi \, R | \le C \epsilon$ on $\text{ supp } \rho$ so that we neglect the corresponding term when now deriving the spatial discretization from (34).

In what follows we assume that $\varOmega$ is polyhedral and consider a family $({\mathcal {T}}_h)_{0 < h \le h_0}$ of triangulations of $\varOmega$ with mesh size $h= \max _{T \in {\mathcal {T}}_h}h_T, \; h_T=\text{ diam }(T)$. We assume that the family is regular in the sense that there exists $\sigma >0$ with

35 $$\begin{aligned} r_T \ge \sigma h_T \qquad \forall T \in {\mathcal {T}}_h \quad \forall 0 < h \le h_0, \end{aligned}$$

where $r_T$ is the radius of the largest ball contained in *T*. Let us denote by ${\mathcal {N}}_h$ the set of vertices of the triangulation ${\mathcal {T}}_h$. In order to formulate our scheme we require a second phase field function with a slightly larger support, namely

$$\begin{aligned} \tilde{\rho }(x,t)=g \left( \frac{\phi (x,t)}{2 \epsilon } \right) , \quad 0<\epsilon < \frac{\delta }{\pi }. \end{aligned}$$

For $0 \le m \le M$ we then define

$$\begin{aligned} {\mathcal {T}}^m_h:= \lbrace T \in {\mathcal {T}}_h \, | \, \tilde{\rho }^m(x)>0 \text{ for } \text{ some } x \in T \cap {\mathcal {N}}_h \rbrace \quad \text{ and } \quad D^m_h:= \bigcup _{T \in {\mathcal {T}}^m_h} T \end{aligned}$$

as well as the finite element space

$$\begin{aligned} V^m_h:= \lbrace v_h \in C^0(D^m_h) \, | \, v_{h|T} \text{ is } \text{ a } \text{ linear } \text{ polynomial } \text{ on } \text{ each } T \in {\mathcal {T}}^m_h \rbrace . \end{aligned}$$

We denote by $I^m_h: C^0(D^m_h) \rightarrow V^m_h$ the standard Lagrange interpolation operator, i.e. $[I^m_h f](x)=f(x), x \in D^m_h \cap {\mathcal {N}}_h$. Note that $D^m_h = \text{ supp } I^m_h \tilde{\rho }^m$.

#### Lemma 3

Suppose that

36 $$\begin{aligned} \displaystyle h \le \frac{\cos ^2\left( \frac{3 \pi }{8}\right) }{2 c_1} \epsilon , \, \tau \le \frac{\cos ^2\left( \frac{3 \pi }{8}\right) }{2 c_2} \epsilon . \end{aligned}$$

Then

- $$U_{\frac{3 \epsilon \pi }{4}}(t) \subset D^m_h \subset U_{\frac{3 \epsilon \pi }{2}}(s)$$ for all $$s, t \in [\max (t_{m-1},0),\min (t_{m+1},T)], 0 \le m \le M$$;
- $$[I^m_h \tilde{\rho }^m](x) \ge \frac{1}{2} \cos ^2\left( \frac{3 \pi }{8}\right) , \, x \in U_{\frac{3 \epsilon \pi }{4}}(t_m), 0 \le m \le M$$.

#### Proof

a) Let $x \in D^m_h$, so that there exists $y \in {\mathcal {N}}_h$ such that $|y-x| \le h$ and $\tilde{\rho }^m(y)>0$. Hence $| \phi ^m(y) | < \epsilon \pi$ and the mean value theorem together with (8) yields for $s \in [\max (t_{m-1},0),\min (t_{m+1},T)]$

$$\begin{aligned} | \phi (x,s) |\le & {} | \phi (x,s) - \phi ^m(x) | + | \phi ^m(x) - \phi ^m(y) | + | \phi ^m(y) | \\< & {} | \phi _t(x,\xi ) | \, | s-t_m| + | \nabla \phi ^m(\eta ) | \, | x-y | + \epsilon \pi \\\le & {} c_2 \tau + c_1 h + \epsilon \pi \le \cos ^2\left( \frac{3 \pi }{8}\right) \epsilon + \epsilon \pi \le \frac{3 \epsilon \pi }{2} \end{aligned}$$

in view of (36). Hence, $x \in U_{\frac{3 \epsilon \pi }{2}}(s)$. Next, let $x \in U_{\frac{3 \epsilon \pi }{4}}(t)$ for some $t \in [\max (t_{m-1},0),\min (t_{m+1},T)]$. Then $\tilde{\rho }(x,t) \ge \cos ^2(\frac{3 \pi }{8})$ and we obtain similarly as above

$$\begin{aligned} {[}I^m_h \tilde{\rho }^m{]}(x)\ge & {} \tilde{\rho }(x,t) - | \tilde{\rho }(x,t) - \tilde{\rho }^m(x)| - | \tilde{\rho }^m(x) - {[}I^m_h \tilde{\rho }^m{]}(x) | \\\ge & {} \cos ^2\left( \frac{3 \pi }{8}\right) - | \tilde{\rho }_t(x,\xi ) | \, | t - t_m| - h \max _{y \in \overline{U_{\delta }(t_m)}} | \nabla \tilde{\rho }^m(y) | \\\ge & {} \cos ^2\left( \frac{3 \pi }{8}\right) - c_2 \frac{\tau }{2 \epsilon } - c_1 \frac{h}{ 2 \epsilon } \ge \frac{1}{2} \cos ^2\left( \frac{3 \pi }{8}\right) . \end{aligned}$$

In particular, $[I^m_h \tilde{\rho }^m](x)>0$, so that $x \in D^m_h$. Using the above inequality for $t=t_m$ implies b). $\square$

Our finite element approximation of (1), (2) now reads: For $m=1,2,\ldots ,M$ find $u^m_h \in V^m_h$ such that for all $v_h \in V^m_h$

37 $$\begin{aligned}&\int _{\varOmega } u^m_h \, v_h \, \rho ^m \, | \nabla \phi ^m | - \int _{\varOmega } u^{m-1}_h \, v_h \, \rho ^{m-1} \, | \nabla \phi ^{m-1} | + \tau _m \, \int _{\varOmega } (\nabla u^m_h, \nabla v_h) \, \rho ^m \, | \nabla \phi ^m | \nonumber \\&\qquad - \tau _m \, \int _{\varOmega } u^m_h \, ({\varvec{v}}^m,\nabla v_h) \, \rho ^m \, | \nabla \phi ^m | + \gamma \tau _m^2 \, \int _{\varOmega } I^m_h \tilde{\rho }^m ( \nabla u^m_h,\nabla v_h)\nonumber \\&\quad = \tau _m \, \int _{\varOmega } f^{e,m} \, v_h \, \rho ^m \, | \nabla \phi ^m |. \end{aligned}$$

Here, $u^0_h \in V^0_h$ is defined as an $L^2$ projection of $u_0^e(x):=u_0(\tilde{p}(x,0)), x \in U_{\delta }(0)$, more precisely

38 $$\begin{aligned} \displaystyle \int _{D^0_h} u^0_h \, v_h = \int _{D^0_h} u_0^e \, v_h \qquad \forall v_h \in V^0_h. \end{aligned}$$

Furthermore, $f^{e,m}(x):=f(\tilde{p}(x,t_m),t_m), x \in U_{\delta }(t_m), 1 \le m \le M$. The parameter $\gamma >0$ will be chosen in such a way as to ensure existence and stability for the scheme, see Lemma 5 and Theorem 1 below.

#### Remark 2

a) Lemma 3 a) implies that $\text{ supp } \rho ^m, \text{ supp } \rho ^{m-1} \subset D^m_h = \text{ supp } I^m_h \tilde{\rho }^m$, so that all integrals appearing in (37) are taken only over $D^m_h$. In particular, if $f \equiv 0$ we see from the choice $v_h \equiv 1$ on $D^m_h$ that the scheme is mass conserving in the sense that

$$\begin{aligned} \int _\varOmega u^m_h \, \rho ^m \, | \nabla \phi ^m | = \int _\varOmega u^0_h \, \rho ^0 \, | \nabla \phi ^0|, \qquad m=1,\ldots ,M. \end{aligned}$$

b) The term $\gamma \tau _m^2 \, \int _{\varOmega } I^m_h \tilde{\rho }^m ( \nabla u^m_h,\nabla v_h)$ introduces artificial diffusion into the scheme and will play a crucial role in our analyis. A different form of stabilization is used in [16], Section 2.5.

c) Unlike the schemes introduced in [16] our method is not fully practical because we assume that the integrals are evaluated exactly. In Sect. 6 we shall follow [16] in using numerical integration to obtain a fully practical scheme. A nice feature of the resulting method is that the evolution of the hypersurfaces is tracked in a simple way via the evaluation of $\rho$.

In what follows we shall be concerned with the existence, stability and error bounds for (37). The extension of our analysis to the fully practical method mentioned above is currently out of reach and left for future research. However, the test calculations in Sect. 6 show that the parameter choices suggested by the analysis work well also for the fully practical scheme.

#### Lemma 4

There exists $0 < h_1 \le h_0$ such that $D^m_h$ is connected for all $0< h \le h_1$ and $0 \le m \le M$.

#### Proof

To begin, we remark that there exists $0<h_1 \le h_0$ and $\mu >0$ only depending on $\sigma , c_0,c_1, c_2$ such that for every $a \in {\mathcal {N}}_h \cap \overline{U_{\delta }(t)}$ there exists a neighbour $b \in {\mathcal {N}}_h$ with

39 $$\begin{aligned} | \phi (a,t) - \phi (b,t) | \ge \mu h_T \qquad \text{ where } a,b, \in T \end{aligned}$$

for all $t \in [0,T], 0 < h \le h_1$. Since $\varGamma (t_m)$ is connected it is sufficient to show that for every $y \in D^m_h$ there exists $z \in \varGamma (t_m)$ and a path in $D^m_h$ connecting *y* to *z*. Let us fix $y \in D^m_h$, say $y \in T$, where $\tilde{\rho }^m(x)>0$ for some $x \in T \cap {\mathcal {N}}_h$. We assume w.l.o.g. that $0< \phi ^m(x) < \epsilon \pi$. In view of (39) there exists a neighbour $x_1 \in {\mathcal {N}}_h$ of *x* such that $\phi ^m(x_1) \le \phi ^m(x) - \mu h_{\tilde{T}}$, where $x,x_1 \in \tilde{T}$. If $\phi ^m(x_1) \le 0$ then there is $z \in [x,x_1]$ with $\phi ^m(z)=0$. Hence, $z \in \varGamma (t_m)$ and the union of the segments [*y*, *x*] and [*x*, *z*] is a path in $D^m_h$ connecting *y* to *z*. If $\phi ^m(x_1)>0$, then $\tilde{\rho }^m(x_1)>0$ so that $[x,x_1] \subset D^m_h$ and we may repeat the above argument with *x* replaced by $x_1$ and so on, until we reach $\varGamma (t_m)$ in a finite number of steps. $\square$

#### Lemma 5

(Existence) Let $0 < h \le h_1$. There exists $\tau _0>0$ such that the scheme (37) has a unique solution $u^m_h \in V^m_h$ provided that $0 < \tau \le \tau _0$.

#### Proof

Since (37) is equivalent to solving a linear system with a quadratic coefficient matrix, it is sufficient to prove that the following problem only has the trivial solution: find $u_h \in V^m_h$ such that for all $v_h \in V^m_h$

$$\begin{aligned}&\int _{\varOmega } u_h \, v_h \, \rho ^m \, | \nabla \phi ^m | + \tau _m \int _{\varOmega } (\nabla u_h, \nabla v_h) \, \rho ^m \, | \nabla \phi ^m | - \tau _m \int _{\varOmega } u_h \, ({\varvec{v}}^m,\nabla v_h) \, \rho ^m \, | \nabla \phi ^m | \\&\quad + \gamma \tau _m^2 \, \int _{\varOmega } I^m_h \tilde{\rho }^m ( \nabla u_h, \nabla v_h) =0. \end{aligned}$$

Inserting $v_h=u_h$ we infer

$$\begin{aligned}&\int _{\varOmega } (u_h)^2 \rho ^m \, | \nabla \phi ^m | + \tau _m \int _{\varOmega } | \nabla u_h |^2 \, \rho ^m \, | \nabla \phi ^m | + \gamma \tau _m^2 \, \int _{\varOmega } I^m_h \tilde{\rho }^m | \nabla u_h |^2 \\&\quad = \tau _m \int _{\varOmega } u_h \, ({\varvec{v}}^m,\nabla u_h) \, \rho ^m \, | \nabla \phi ^m | \le \tau _m \, \max _{x \in \overline{U_{\delta }(t_m)}} | {\varvec{v}}^m(x) | \, \int _\varOmega | u_h | \, | \nabla u_h | \, \rho ^m \, | \nabla \phi ^m | \\&\quad \le \frac{1}{2} \int _{\varOmega } (u_h)^2 \, \rho ^m \, | \nabla \phi ^m | + \frac{1}{2} \tau \, \left( \max _{x \in \overline{U_{\delta }(t_m)}} | {\varvec{v}}^m(x) |\right) ^2 \tau _m \int _{\varOmega } | \nabla u_h |^2 \, \rho ^m \, | \nabla \phi ^m |. \end{aligned}$$

If we choose $\tau _0>0$ so small that $\frac{1}{2} \tau \, \bigl ( \max _{x \in \overline{U_{\delta }(t_m)}} | {\varvec{v}}^m(x) | \bigr )^2 \le 1$ we deduce that

$$\begin{aligned} \int _{\varOmega } (u_h)^2 \, \rho ^m \, | \nabla \phi ^m | = \int _{\varOmega } I^m_h \tilde{\rho }^m | \nabla u_h |^2=0, \end{aligned}$$

which implies that $u_h \equiv 0$ on $\varGamma (t_m)$ and $\nabla u_h \equiv 0$ in $D^m_h$. According to Lemma 4, $D^m_h$ is connected, so that we conclude that $u_h \equiv 0$. $\square$

## Stability bound

The following lemma will be useful in estimating $L^2$-integrals that are not weighted by $\rho$.

### Lemma 6

There exists $C\ge 0$ such that for $t \in [0,T]$:

40 $$\begin{aligned} \int _{U_{\frac{3 \epsilon \pi }{4}}(t)} f^2 \le C \int _\varOmega f^2 \rho (\cdot ,t) | \nabla \phi (\cdot ,t)| +C\varepsilon ^2\int _{U_{\frac{3 \epsilon \pi }{4}}(t)} | \nabla f |^2 \qquad \text{ for } \text{ all } f \in H^1(\varOmega ). \end{aligned}$$

### Remark 3

Note that Lemma 3 b) implies that

41 $$\begin{aligned} \displaystyle \int _{U_{\frac{3 \epsilon \pi }{4}}(t_m)} | \nabla f |^2 \le \frac{2}{\cos ^2(\frac{3 \pi }{8})} \int _\varOmega I^m_h \tilde{\rho }^m | \nabla f |^2, \quad f \in H^1(\varOmega ), m=0,\ldots ,M. \end{aligned}$$

### Proof

We may assume that *f* is smooth, the general case then follows with the help of an approximation argument. Since $F_t$ is a diffeomorphism from $\varGamma (0) \times (-\frac{3 \epsilon \pi }{4}, \frac{3 \epsilon \pi }{4})$ onto $U_{\frac{3 \epsilon \pi }{4}}(t)$, the transformation rule yields

42 $$\begin{aligned} \displaystyle c_1 \int _{U_{\frac{3\epsilon \pi }{4}}(t)} f(x)^2 dx \le \int _{-\frac{3 \varepsilon \pi }{4}}^{\frac{3 \varepsilon \pi }{4}}\int _{\varGamma (0)} f(F_t(P,s))^2 do_P ds \le c_2 \int _{U_{\frac{3\epsilon \pi }{4}}(t)} f(x)^2 dx. \end{aligned}$$

The definition of $F_t$ together with (9) implies for $|s|\le \frac{3 \varepsilon \pi }{4}$, $|\tilde{s} | \le \frac{\varepsilon \pi }{4}$

$$\begin{aligned} f(F_t(P,s))= & {} f(F_t(P,\tilde{s}))+ \int _{\tilde{s}}^s \left( \nabla f(F_t(P,r)), \frac{\partial F_t}{\partial r}(P,r) \right) dr \\= & {} f(F_t(P,\tilde{s}))+\int _{\tilde{s}}^s \left( \nabla f(F_t(P,r)), \frac{\nabla \phi (F_t(P,r),t)}{|\nabla \phi (F_t(P,r),t)|^2}\right) dr \end{aligned}$$

and therefore

$$\begin{aligned} f(F_t(P,s))^2\le & {} 2 f(F_t(P,\tilde{s}))^2 + C \varepsilon \int _{-\frac{3 \varepsilon \pi }{4}}^{\frac{3 \varepsilon \pi }{4}} | \nabla f (F_t(p,r)) |^2 dr \\\le & {} C f(F_t(P,\tilde{s}))^2 \rho (F_t(P,\tilde{s}),t) +C\varepsilon \int _{-\frac{3 \varepsilon \pi }{4}}^{\frac{3 \varepsilon \pi }{4}} | \nabla f (F_t(p,r)) |^2 dr, \end{aligned}$$

since $\rho (F_t(P,\tilde{s}),t) = \cos ^2 \bigl (\frac{\phi (F_t(P,\tilde{s}),t)}{\epsilon } \bigr ) = \cos ^2(\frac{\tilde{s}}{\epsilon }) \ge \cos ^2(\frac{\pi }{4}), | \tilde{s} | \le \frac{\epsilon \pi }{4}$. Integrating with respect to $P \in \varGamma (0), s \in (-\frac{3 \varepsilon \pi }{4},\frac{3 \varepsilon \pi }{4})$ and recalling (42) we obtain for $|\tilde{s} | \le \frac{\varepsilon \pi }{4}$

$$\begin{aligned} \int _{U_{\frac{3 \epsilon \pi }{4}}(t)} f(x)^2 dx\le & {} C \epsilon \int _{\varGamma (0)} f(F_t(P,\tilde{s}))^2 \rho (F_t(P,\tilde{s}),t) do_P \\&+ C \epsilon ^2 \int _{-\frac{3 \varepsilon \pi }{4}}^{\frac{3 \varepsilon \pi }{4}} \int _{\varGamma (0)} | \nabla f (F_t(p,r)) |^2 do_P dr \\\le & {} C \epsilon \int _{\varGamma (0)} f(F_t(P,\tilde{s}))^2 \rho (F_t(P,\tilde{s}),t) do_P \\&+ C\varepsilon ^2 \int _{U_{\frac{3 \epsilon \pi }{4}}(t)} | \nabla f(x) |^2 dx. \end{aligned}$$

If we integrate with respect to $\tilde{s} \in (-\frac{\varepsilon \pi }{4},\frac{\varepsilon \pi }{4})$, divide by $\epsilon$ and recall (8) we obtain the assertion. $\square$

It follows from Theorem 4.4 in [8] (extended in a straightforward way to the case of a nontrivial *f*) that (1), (2) has a unique solution *u* which satisfies

$$\begin{aligned}&\sup _{(0,T)} \Vert u(\cdot ,t) \Vert _{L^2(\varGamma (t))}^2 + \int _0^T \Vert \nabla _{\varGamma } u(\cdot ,t) \Vert _{L^2(\varGamma (t))}^2 dt \le c \left( \Vert u_0 \Vert _{L^2(\varGamma (0))}^2 \right. \\&\left. \quad + \int _0^T \Vert f(\cdot ,t) \Vert _{L^2(\varGamma (t))}^2 dt \right) . \end{aligned}$$

The following theorem gives a discrete version of this estimate in the phase field setting.

### Theorem 1

Suppose that (36) holds. There exist $\gamma _1 >0$ and $\tau _1 \le \tau _0$ such that

$$\begin{aligned}&\max _{m=1,\ldots ,M} \frac{2}{\epsilon \pi } \int _{\varOmega } (u^m_h)^2 \, \rho ^m \, | \nabla \phi ^m | + \, \sum _{m=1}^M \tau _m \, \frac{2}{\epsilon \pi } \int _{\varOmega } | \nabla u^m_h |^2 \rho ^m \, | \nabla \phi ^m | \\&\quad \le C \left( \int _{\varGamma (0)} (u_0)^2 + \sum _{m=1}^M \tau _m \int _{\varGamma (t_m)} (f^m)^2 \right) , \end{aligned}$$

provided that $\gamma \ge \gamma _1$ and $\tau \le \min \bigl ( \tau _1, \epsilon ^2 \bigr )$.

### Proof

Setting $v_h=u^m_h$ in (37) we find after a straighforward calculation

43 $$\begin{aligned}&\frac{1}{2}\int _\varOmega (u^m_h)^2 \, \rho ^m\, |\nabla \phi ^{m}|- \frac{1}{2}\int _\varOmega (u^{m-1}_h)^2 \, \rho ^{m-1}\, | \nabla \phi ^{m-1}|\nonumber \\&\qquad +\frac{1}{2}\int _\varOmega (u^m_h-u^{m-1}_h)^2 \, \rho ^{m-1}\, | \nabla \phi ^{m-1}|\nonumber \\&\qquad + \tau _m \int _\varOmega |\nabla u^m_h|^2 \, \rho ^m\, |\nabla \phi ^{m}|+ \gamma \tau _m^2 \int _\varOmega I^m_h \tilde{\rho }^m | \nabla u^m_h|^2 \nonumber \\&\quad = - \frac{1}{2}\int _\varOmega (u^m_h)^2 \, (\rho ^m-\rho ^{m-1}) \, | \nabla \phi ^{m-1}|+ \frac{1}{2}\int _\varOmega (u^m_h)^2 \, \rho ^m\, (| \nabla \phi ^{m-1}|-|\nabla \phi ^{m}|)\nonumber \\&\qquad + \tau _m \int _\varOmega u^m_h({\varvec{v}}^{m},\nabla u^m_h) \, \rho ^m\, |\nabla \phi ^{m}|+ \tau _m \int _\varOmega f^{e,m} u^m_h\, \rho ^m\, |\nabla \phi ^{m}|\nonumber \\&\quad := I + II + III + IV. \end{aligned}$$

Clearly,

44 $$\begin{aligned} I = - \frac{1}{2}\int _{t_{m-1}}^{t_m} \int _\varOmega (u^m_h)^2 \, \rho _t(\cdot ,s) \, | \nabla \phi ^{m-1}|ds, \end{aligned}$$

while

45 $$\begin{aligned} II= & {} - \frac{1}{2}\tau _m \int _\varOmega (u^m_h)^2 \, \rho ^m\, (\nabla \phi _t^m, \nu ^m)\nonumber \\&+\frac{1}{2} \int _\varOmega (u^m_h)^2 \, \rho ^m\, \bigl (| \nabla \phi ^{m-1}|- |\nabla \phi ^{m}|+\tau _m (\nabla \phi ^m_t,\nu ^m) \bigr ) = II_1 + II_2. \end{aligned}$$

Integrating by parts and abbreviating $H^m = - \nabla \cdot \nu ^m$ we obtain

$$\begin{aligned} II_1= & {} \frac{1}{2} \tau _m \int _\varOmega (u^m_h)^2 \, (\nabla \rho ^m, \nu ^m) \, \phi _t^m + \tau _m \int _\varOmega u^m_h(\nabla u^m_h,\nu ^m) \rho ^m\, \phi _t^m\\&+ \frac{1}{2} \tau _m \int _\varOmega (u^m_h)^2 \, \nabla \cdot \nu ^m \, \rho ^m\, \phi _t^m \\= & {} \frac{1}{2} \tau _m \int _\varOmega (u^m_h)^2 \, \rho _t^m \, |\nabla \phi ^{m}|+ \tau _m \int _\varOmega u^m_h(\nabla u^m_h,\nu ^m) \rho ^m\, \phi _t^m \\&- \frac{1}{2} \tau _m \int _\varOmega (u^m_h)^2 H^m \, \rho ^m\, \phi _t^m, \end{aligned}$$

since

$$\begin{aligned} ( \nabla \rho ^m, \nu ^m) \phi ^m_t = \frac{1}{\epsilon } g'\left( \frac{\phi ^m}{\epsilon }\right) \, \phi ^m_t \, ( \nabla \phi ^m,\nu ^m) = \rho ^m_t \, | \nabla \phi ^m |. \end{aligned}$$

In order to rewrite *III* we first observe that in view of (22) and (24)

$$\begin{aligned} ({\varvec{v}}^{m}, \nabla u^m_h)= & {} ({\varvec{v}}^{m}, \nabla _{\phi ^m} u^m_h) + (\nabla u^m_h, \nu ^m) ({\varvec{v}}^{m}, \nu ^m) \\= & {} ({\varvec{v}}^{m}, \nabla _{\phi ^m} u^m_h) - (\nabla u^m_h, \nu ^m) \frac{ \phi ^m_t}{|\nabla \phi ^{m}|}, \end{aligned}$$

so that (23), (25) and again (24) imply

46 $$\begin{aligned} III= & {} \frac{1}{2} \tau _m \int _\varOmega ({\varvec{v}}^{m}, \nabla _{\phi ^m}(u^m_h)^2) \rho ^m\, |\nabla \phi ^{m}|- \tau _m \int _\varOmega u^m_h(\nabla u^m_h, \nu ^m) \rho ^m\, \phi _t^m \nonumber \\= & {} - \frac{1}{2} \tau _m \int _\varOmega \nabla _{\phi ^m } \cdot {\varvec{v}}^{m}(u^m_h)^2 \, \rho ^m\, |\nabla \phi ^{m}|- \frac{1}{2} \tau _m \int _\varOmega H^m ( {\varvec{v}}^{m}, \nu ^m) (u^m_h)^2 \, \rho ^m\, |\nabla \phi ^{m}|\nonumber \\&- \tau _m \int _\varOmega u^m_h(\nabla u^m_h, \nu ^m) \rho ^m\, \phi _t^m = - \frac{1}{2} \tau _m \int _\varOmega \nabla _{\phi ^m} \cdot {\varvec{v}}^{m}(u^m_h)^2 \, \rho ^m\, |\nabla \phi ^{m}|\nonumber \\&+ \frac{1}{2} \tau _m \int _\varOmega (u^m_h)^2H^m \, \rho ^m\, \phi _t^m - \tau _m \int _\varOmega u^m_h(\nabla u^m_h, \nu ^m) \, \rho ^m\, \phi _t^m. \end{aligned}$$

Inserting (44)–(46) into (43) we infer that

47 $$\begin{aligned}&\frac{1}{2}\int _\varOmega (u^m_h)^2 \, \rho ^m\,|\nabla \phi ^{m}|- \frac{1}{2}\int _\varOmega (u^{m-1}_h)^2 \, \rho ^{m-1}\, | \nabla \phi ^{m-1}|+ \tau _m \int _\varOmega |\nabla u^m_h|^2 \, \rho ^m\, |\nabla \phi ^{m}|\nonumber \\&\quad + \gamma \tau _m^2 \int _\varOmega I^m_h \tilde{\rho }^m | \nabla u^m_h|^2 \quad \le \frac{1}{2}\int _{t_{m-1}}^{t_m}\int _\varOmega (u^m_h)^2 \left( \rho _t^m |\nabla \phi ^{m}|-\rho _t(.,s)| \nabla \phi ^{m-1}|\right) \nonumber \\&\quad -\frac{1}{2} \tau _m \int _\varOmega \nabla _{\phi ^m}\cdot {\varvec{v}}^{m}(u^m_h)^2 \, \rho ^m\, |\nabla \phi ^{m}|+ \frac{1}{2} \int _\varOmega (u^m_h)^2 \, \rho ^m\, \bigl ( | \nabla \phi ^{m-1}|\nonumber \\&\quad - |\nabla \phi ^{m}|+\tau _m (\nabla \phi ^m_t,\nu ^m) \bigr ) + \tau _m \int _\varOmega f^{e,m} u^m_h\, \rho ^m\,|\nabla \phi ^{m}|. \end{aligned}$$

We deduce from (28), Lemma 6, (41) and the assumption $\tau \le \epsilon ^2$ that

$$\begin{aligned}&\left| \frac{1}{2} \int _{t_{m-1}}^{t_m}\int _\varOmega (u^m_h)^2 (\rho ^m_t|\nabla \phi ^{m}|-\rho _t(.,s)| \nabla \phi ^{m-1}|) \right| \\&\quad \le C \int _{t_{m-1}}^{t_m}\int _\varOmega (u^m_h)^2 \bigl ( | \rho ^m_t - \rho _t(.,s) | + | \rho _t(.,s) | \, | |\nabla \phi ^{m}|-| \nabla \phi ^{m-1}|| \bigr ) \\&\quad \le C \frac{\tau _m^2}{\epsilon ^2} \int _{U_{\frac{3 \epsilon \pi }{4}}(t_m)} (u^m_h)^2 \le C \frac{\tau _m^2}{\epsilon ^2} \int _\varOmega (u^m_h)^2 \, \rho ^m|\nabla \phi ^{m}|+ C \tau _m^2 \int _{U_{\frac{3 \epsilon \pi }{4}}(t_m)} | \nabla u^m_h|^2\\&\quad \le C \tau _m \int _\varOmega (u^m_h)^2 \, \rho ^m|\nabla \phi ^{m}|+ (\gamma -1) \tau _m^2 \int _\varOmega I_h \tilde{\rho }^m \, | \nabla u^m_h|^2 \end{aligned}$$

if we choose $\gamma \ge \gamma _1:=C+1$. Finally, using Taylor expansion and (8) we infer that

$$\begin{aligned}&\left| -\frac{1}{2} \tau _m \int _\varOmega \nabla _{\phi ^m}\cdot {\varvec{v}}^{m}(u^m_h)^2 \, \rho ^m\, |\nabla \phi ^{m}|\right. \\&\quad \left. + \frac{1}{2} \int _\varOmega (u^m_h)^2 \, \rho ^m\, \bigl ( | \nabla \phi ^{m-1}|- |\nabla \phi ^{m}|+\tau _m (\nabla \phi ^m_t,\nu ^m) \bigr ) \right| \\&\quad \le C \tau _m \int _\varOmega (u^m_h)^2 \, \rho ^m|\nabla \phi ^{m}|+ C \tau _m^2 \int (u^m_h)^2 \rho ^m \le C \tau _m \int _\varOmega (u^m_h)^2 \rho ^m |\nabla \phi ^{m}|. \end{aligned}$$

Inserting the above estimates into (47) we find

48 $$\begin{aligned}&\frac{1}{2}\int _\varOmega (u^m_h)^2 \, \rho ^m\, |\nabla \phi ^{m}|+ \tau _m \int _\varOmega |\nabla u^m_h|^2 \, \rho ^m\, |\nabla \phi ^{m}|+ \tau _m^2 \int _\varOmega I^m_h \tilde{\rho }^m | \nabla u^m_h|^2 \nonumber \\&\qquad \le \frac{1}{2}\int _\varOmega (u^{m-1}_h)^2 \, \rho ^{m-1}\, | \nabla \phi ^{m-1}|+ C \tau _m \int _\varOmega (u^m_h)^2 \, \rho ^m \, |\nabla \phi ^{m}|\nonumber \\&\qquad + \tau _m \int _\varOmega (f^{e,m})^2 \, \rho ^m \, |\nabla \phi ^{m}|. \end{aligned}$$

If $\tau _1 \le \tau _0$ is sufficiently small we therefore deduce for $\tau \le \tau _1$

$$\begin{aligned}&\int _\varOmega (u^m_h)^2 \, \rho ^m\, |\nabla \phi ^{m}|+ \tau _m \int _\varOmega |\nabla u^m_h|^2 \, \rho ^m\, |\nabla \phi ^{m}|\\&\quad \le (1+ C \tau _m) \int _\varOmega (u^{m-1}_h)^2 \, \rho ^{m-1}\, | \nabla \phi ^{m-1}|+ C \tau _m \int _\varOmega (f^{e,m})^2 \, \rho ^m \, |\nabla \phi ^{m}|, \end{aligned}$$

from which we obtain after summation from $m=1,\ldots ,l$ and division by $\epsilon$ that

$$\begin{aligned}&\frac{1}{\epsilon } \int _\varOmega (u_h^l)^2 \, \rho ^l \, | \nabla \phi ^l | + \sum _{m=1}^l \tau _m \frac{1}{\epsilon } \int _\varOmega |\nabla u^m_h|^2 \, \rho ^m\, |\nabla \phi ^{m}|\\&\quad \le \frac{1}{\epsilon } \int _\varOmega (u_h^0)^2 \, \rho ^0 \, | \nabla \phi ^0 | + C \sum _{m=0}^{l-1} \tau _{m+1} \frac{1}{\epsilon } \int _\varOmega (u^m_h)^2 \, \rho ^m\, |\nabla \phi ^{m}|\\&\quad +C \sum _{m=1}^l \tau _m \frac{1}{\epsilon } \int _\varOmega (f^{e,m})^2 \, \rho ^m \, |\nabla \phi ^{m}|. \end{aligned}$$

Using Lemma 3 a), (38) and (16) we may estimate

$$\begin{aligned} \frac{1}{\epsilon } \int _\varOmega (u_h^0)^2 \, \rho ^0 \, | \nabla \phi ^0 |\le & {} \frac{C}{\epsilon } \int _{D^0_h} (u^0_h)^2 \le \frac{C}{\epsilon } \int _{D^0_h} (u^e_0)^2 \le \frac{C}{\epsilon } \int _{U_{\frac{3 \epsilon \pi }{2}}(0)} (u^e_0)^2\\\le & {} C \int _{\varGamma (0)} (u_0)^2. \end{aligned}$$

Arguing in a similar way for the term involving $f^{e,m}$ we derive

49 $$\begin{aligned}&\frac{1}{\epsilon } \int _\varOmega (u_h^l)^2 \, \rho ^l \, | \nabla \phi ^l | + \sum _{m=1}^l \tau _m \frac{1}{\epsilon } \int _\varOmega |\nabla u^m_h|^2 \, \rho ^m\, |\nabla \phi ^{m}|\nonumber \\&\quad \le C \sum _{m=0}^{l-1} \tau _{m+1} \frac{1}{\epsilon } \int _\varOmega (u^m_h)^2 \, \rho ^m\, |\nabla \phi ^{m}|+ C \left( \int _{\varGamma (0)} (u_0)^2\right. \nonumber \\&\left. \quad + \sum _{m=1}^l \tau _m \int _{\varGamma (t_m)} (f^m)^2 \right) . \end{aligned}$$

The discrete Gronwall inequality yields the bound on $\max _{m=1,\ldots ,M} \frac{1}{\epsilon } \int _{\varOmega } (u^m_h)^2 \, \rho ^m \, | \nabla \phi ^m |$, which combined with (49) implies the second inequality. $\square$

## Error estimate

Before we formulate our error bound we derive interpolation estimates that are adapted to our setting.

### Lemma 7

Suppose that (36) holds and let $z^e$ be defined by (14). Then we have for $m=1,\ldots ,M$ and $t \in [t_{m-1},t_m]$:

$$\begin{aligned}&\int _{D^m_h} | (z^e - I^m_h z^e)(\cdot ,t) |^2 + h^2 \int _{D^m_h} | \nabla (z^e - I^m_h z^e)(\cdot ,t) |^2 \le C \epsilon h^4 \Vert z(\cdot ,t) \Vert _{H^2(\varGamma (t))}^2, \\&\quad \int _{D^m_h} | (z^e_t - I^m_h z^e_t)(\cdot ,t) |^2 \le C \epsilon h^4 \bigl ( \Vert \partial _t^{\bullet } z(\cdot ,t) \Vert _{H^2(\varGamma (t))}^2 + \Vert z(\cdot ,t) \Vert _{H^3(\varGamma (t))}^2 \bigr ). \end{aligned}$$

### Proof

Let $t \in [t_{m-1},t_m]$. Standard interpolation theory together with Lemma 3 a) and (16) implies that

$$\begin{aligned}&\int _{D^m_h} | (z^e - I^m_h z^e)(\cdot ,t) |^2 + h^2 \int _{D^m_h} | \nabla (z^e - I^m_h z^e)(\cdot ,t) |^2 \\&\quad \le c h^4 \int _{D^m_h} | D^2 z^e(\cdot ,t) |^2 \le c h^4 \int _{U_{\frac{3 \epsilon \pi }{2}}(t)} | D^2 z^e(\cdot ,t) |^2 \le C \epsilon h^4 \Vert z(\cdot ,t) \Vert _{H^2(\varGamma (t))}^2. \end{aligned}$$

The second bound follows in the same way using (17). $\square$

### Theorem 2

Suppose that the solution of (1), (2) satisfies

50 $$\begin{aligned} \displaystyle \max _{t \in [0,T]} \Vert u(\cdot ,t) \Vert _{W^{2,\infty }(\varGamma (t))}^2 + \int _0^T \bigl ( \Vert u(\cdot ,t) \Vert _{H^3(\varGamma (t))}^2 + \Vert \partial _t^{\bullet } u(\cdot ,t) \Vert _{H^2(\varGamma (t))}^2 \bigr ) dt < \infty . \end{aligned}$$

Then there exists $0< \tau _2 \le \tau _1$ and a constant $C\ge 0$ such that

$$\begin{aligned}&\max _{m=1,\ldots ,M} \frac{2}{\epsilon \pi } \int _{\varOmega } \, | u^{e,m} - u^m_h|^2 \, \rho ^m \, | \nabla \phi ^m | \\&\quad + \sum _{m=1}^M \tau _m \frac{2}{\epsilon \pi } \int _{\varOmega } | \nabla (u^{e,m}- u^m_h) |^2 \rho ^m \, | \nabla \phi ^m | \le C \epsilon ^2, \end{aligned}$$

provided that $\tau \le \min (\epsilon ^2,\tau _2)$, $\gamma \ge \gamma _1$ and (36) hold.

### Proof

Let us write

$$\begin{aligned} u^{e,m} - u^m_h = (u^{e,m} - I^m_h u^{e,m}) + (I^m_h u^{e,m} - u^m_h)=: d^m + e^m_h. \end{aligned}$$

If we combine (34) for $\eta = v_h \in V^m_h$ with (37) we find

$$\begin{aligned}&\int _\varOmega e_h^{m}v_h \, \rho ^m|\nabla \phi ^{m}|-\int _\varOmega e_h^{m-1}v_h \, \rho ^{m-1}| \nabla \phi ^{m-1}|+ \tau _m \int _\varOmega (\nabla e_h^{m},\nabla v_h) \rho ^m|\nabla \phi ^{m}|\\&\qquad -\tau _m \int _\varOmega e_h^{m}({\varvec{v}}^{m},\nabla v_h) \rho ^m|\nabla \phi ^{m}|+ \gamma \tau _m^2\int _\varOmega I^m_h \tilde{\rho }^m (\nabla e_h^{m},\nabla v_h) \\&\quad = \left[ - \int _\varOmega d^{m}v_h \, \rho ^m|\nabla \phi ^{m}|+ \int _\varOmega d^{m-1}v_h \, \rho ^{m-1}| \nabla \phi ^{m-1}|\right] \\&\qquad - \tau _m \int _\varOmega (\nabla d^{m},\nabla v_h) \rho ^m|\nabla \phi ^{m}|+ \tau _m \int _\varOmega d^{m}({\varvec{v}}^{m},\nabla v_h) \rho ^m|\nabla \phi ^{m}|\\&\qquad + \gamma \tau _m^2\int _\varOmega I^m_h \tilde{\rho }^m (\nabla I_h u^{e,m},\nabla v_h) + \int _{t_{m-1}}^{t_m}\int _\varOmega \left[ (\nabla u^{e,m}, \nabla v_h) \rho ^m|\nabla \phi ^{m}|\right. \\&\qquad \left. - (\nabla u^e,\nabla v_h) \rho \, | \nabla \phi |\right] \! +\!\! \int _{t_{m-1}}^{t_m}\int _\varOmega \left[ u^e ({\varvec{v}}, \nabla v_h) \rho \, | \nabla \phi |- \! u^{e,m} ({\varvec{v}}^{m}, \nabla v_h) \rho ^m|\nabla \phi ^{m}|\right] \\&\qquad +\int _{t_{m-1}}^{t_m}\int _\varOmega \left[ f^ev_h \, \rho \, | \nabla \phi |- f^{e,m} v_h \, \rho ^m|\nabla \phi ^{m}|\right] +\int _{t_{m-1}}^{t_m}\int _\varOmega \phi \, R \, v_h \, \rho \, | \nabla \phi |\\&\quad =: \sum _{i=1}^8 \langle S_i^m, v_h \rangle . \end{aligned}$$

Inserting $v_h= e^m_h$ and following the argument in the proof of Theorem 1 leading to (48) we obtain

51 $$\begin{aligned}&\frac{1}{2}\int _\varOmega (e_h^{m})^2 \rho ^m|\nabla \phi ^{m}|+ \tau _m \int _\varOmega |\nabla e_h^{m}|^2 \rho ^m|\nabla \phi ^{m}|+ \tau _m^2 \int _\varOmega I^m_h \tilde{\rho }^m | \nabla e_h^{m}| ^2 \nonumber \\&\quad \le \frac{1}{2}\int _\varOmega (e_h^{m-1})^2 \rho ^{m-1}| \nabla \phi ^{m-1}|+ C \tau _m \int _\varOmega (e_h^{m})^2 \rho ^m |\nabla \phi ^{m}|+ \sum _{i=1}^8 \langle S^m_i,e_h^{m}\rangle .\nonumber \\ \end{aligned}$$

We now deal individually with the terms $\langle S^m_i,e_h^{m}\rangle , i =1,\ldots ,8$ in (51). Clearly,

$$\begin{aligned}&| \langle S_1^m, e_h^{m}\rangle | \le C \int _\varOmega | d^m - d^{m-1} | \, | e_h^{m}| \, \rho ^m+ C \int _\varOmega | d^{m-1} | \, | e_h^{m}| \, | \nabla ( \phi ^m - \phi ^{m-1}) | \, \rho ^m\\&\qquad + C \int _\varOmega | d^{m-1} | \, | e_h^{m}| \, | \rho ^m- \rho ^{m-1}| \equiv I + II + III. \end{aligned}$$

In order to estimate *I* we first deduce from Lemma 3 a) that every $T \in {\mathcal {T}}_h$ with $T \cap \text{ supp } \rho ^m \ne \emptyset$ satisfies $T \in {\mathcal {T}}^{m-1}_h \cap {\mathcal {T}}^m_h$. Therefore $I^{m-1}_h u^{e,m-1} = I^m_h u^{e,m-1}$ on $\text{ supp } \rho ^m$, which yields

$$\begin{aligned} d^m - d^{m-1}= & {} [u^{e,m}- u^{e,m-1}]- I^m_h[u^{e,m}- u^{e,m-1}] \\= & {} \int _{t_{m-1}}^{t_m} (u^e_t - I^m_h u^e_t)(\cdot ,t) \quad \text{ on } \text{ supp } \rho ^m. \end{aligned}$$

Hence, Lemma 3 a), Lemma 7 and (8) imply that

$$\begin{aligned} | I |\le & {} C \int _\varOmega \int _{t_{m-1}}^{t_m} | u^e_t - I^m_h u^e_t | \, | e_h^{m}| \, \rho ^m\\\le & {} C \sqrt{\tau _m} \left( \int _\varOmega (e_h^{m})^2 \rho ^m\right) ^{\frac{1}{2}} \left( \int _{t_{m-1}}^{t_m} \int _{D^m_h} | u^e_t - I^m_h u^e_t |^2 \right) ^{\frac{1}{2}} \\\le & {} \tau _m \int _\varOmega (e^m_h)^2 \rho ^m|\nabla \phi ^{m}|+ C \epsilon h^4 \int _{t_{m-1}}^{t_m} \left( \Vert \partial ^{\bullet }_t u(\cdot ,t) \Vert _{H^2(\varGamma (t))}^2 \right. \\&\left. +\Vert u(\cdot ,t) \Vert _{H^3(\varGamma (t))}^2 \right) dt \end{aligned}$$

and similarly,

$$\begin{aligned} | II |\le & {} C \tau _m \int _\varOmega | d^{m-1} | \, | e_h^{m}| \, \rho ^m\le C \tau _m \left( \int _\varOmega (e_h^{m})^2 \rho ^m\right) ^{\frac{1}{2}} \left( \int _{D^{m-1}_h} | d^{m-1} |^2 \right) ^{\frac{1}{2}} \\\le & {} \tau _m \int _\varOmega (e^m_h)^2 \rho ^m|\nabla \phi ^{m}|+ C \epsilon h^4 \tau _m \Vert u^{m-1} \Vert _{H^2(\varGamma (t_{m-1}))}^2. \end{aligned}$$

Next, we deduce from (27), Lemma 3 a), (8), Lemma 7, Lemma 6 and (41) that

$$\begin{aligned} | III |\le & {} C \frac{\tau _m}{\epsilon } \int _\varOmega | d^{m-1} \, | e_h^{m}| \, \sqrt{\rho ^m} + C \frac{\tau _m^2}{\epsilon ^2} \int _{U_{\frac{3 \epsilon \pi }{4}}(t_m)} | d^{m-1} | \, | e_h^{m}| \\\le & {} \tau _m \int _\varOmega (e_h^{m})^2 \rho ^m + C \frac{\tau _m}{\epsilon ^2} \Vert d^{m-1} \Vert _{L^2(D^{m-1}_h)}^2 \\&\quad + C \frac{\tau _m^2}{\epsilon ^2} \left( \int _{ U_{\frac{3 \epsilon \pi }{4}}(t_m)} ( e_h^{m})^2 \right) ^{\frac{1}{2}} \Vert d^{m-1} \Vert _{L^2(D^{m-1}_h)} \\\le & {} C \tau _m \int _\varOmega (e_h^{m})^2 \rho ^m |\nabla \phi ^{m}|+ C \frac{\tau _m h^4}{\epsilon } \Vert u^{m-1} \Vert _{H^2(\varGamma (t_{m-1}))}^2 \\&\quad + C \frac{\tau _m^2 h^2}{\epsilon ^{\frac{3}{2}}} \Vert u^{m-1} \Vert _{H^2(\varGamma (t_{m-1}))} \left( \int _\varOmega ( e_h^{m})^2 \rho ^m|\nabla \phi ^{m}|\! +\! \epsilon ^2 \int _{ U_{\frac{3 \epsilon \pi }{4}}(t_m)} | \nabla e^m_h |^2 \right) ^{\frac{1}{2}} \\\le & {} C \tau _m \int _\varOmega (e^m_h)^2 \rho ^m|\nabla \phi ^{m}|+ \frac{\tau _m^2}{8} \int _\varOmega I^m_h \tilde{\rho }^m | \nabla e^m_h |^2 \!+\! C \frac{\tau _m h^4}{\epsilon } \Vert u^{m-1} \Vert _{H^2(\varGamma (t_{m-1}))}^2, \end{aligned}$$

where we used that $\tau \le \epsilon ^2$. Again by Lemma 7 we have

$$\begin{aligned} | \langle S^m_2,e_h^{m}\rangle |\le & {} \tau _m \left( \int _\varOmega | \nabla e_h^{m}|^2 \rho ^m|\nabla \phi ^{m}|\right) ^{\frac{1}{2}} \left( \int _{D^m_h} | \nabla d^m |^2 \right) ^{\frac{1}{2}} \\\le & {} \frac{1}{8} \tau _m \int _\varOmega | \nabla e_h^{m}|^2 \rho ^m|\nabla \phi ^{m}|+ C \tau _m \epsilon h^2 \Vert u^m \Vert _{H^2(\varGamma (t_m))}^2, \end{aligned}$$

while

$$\begin{aligned} | \langle S^m_3,e_h^{m}\rangle |\le & {} C \tau _m \int _\varOmega | d^m | \, | \nabla e_h^{m}| \, \rho ^m|\nabla \phi ^{m}|\\\le & {} C \tau _m \left( \int _\varOmega | \nabla e_h^{m}|^2 \rho ^m|\nabla \phi ^{m}|\right) ^{\frac{1}{2}} \left( \int _{D^m_h} | d^m|^2 \right) ^{\frac{1}{2}} \\\le & {} \frac{1}{8} \tau _m \int _\varOmega | \nabla e_h^{m}|^2 \rho ^m|\nabla \phi ^{m}|+ C \tau _m \epsilon h^4 \Vert u^m \Vert _{H^2(\varGamma (t_m))}^2. \end{aligned}$$

Lemma 3 a), (16) and Lemma 7 yield

$$\begin{aligned} | \langle S^m_4,e_h^{m}\rangle |\le & {} C \tau _m^2 \left( \int _\varOmega I^m_h \tilde{\rho }^m | \nabla e_h^{m}|^2 \right) ^{\frac{1}{2}} \left( \int _{D^m_h} | \nabla I^m_h u^{e,m} |^2 \right) ^{\frac{1}{2}} \\\le & {} \frac{\tau _m^2}{8} \int _\varOmega I^m_h \tilde{\rho }^m | \nabla e_h^{m}|^2 + C \tau _m^2 \int _{D^m_h} \bigl ( | \nabla u^{e,m} |^2 + | \nabla d^m |^2 \bigr ) \\\le & {} \frac{\tau _m^2}{8} \int _\varOmega I^m_h \tilde{\rho }^m | \nabla e_h^{m}|^2 + C \tau _m^2 \epsilon \Vert u^m \Vert _{H^2(\varGamma (t_m))}^2. \end{aligned}$$

We deduce from (27), Lemma 3 a), Lemma 1 and (41) that

$$\begin{aligned}&| \langle S^m_5,e_h^{m}\rangle | \le C \int _{t_{m-1}}^{t_m} \int _\varOmega \left[ | \nabla (u^{e,m} - u^e) | \, \rho ^m \right. \\&\qquad \left. + | \nabla u^e | \, | \nabla (\phi ^m - \phi ) | \, \rho ^m + | \nabla u^e | \, | \rho ^m - \rho | \right] | \nabla e_h^{m}| \\&\quad \le C \tau _m \int _{t_{m-1}}^{t_m} \int _\varOmega | \nabla u^e_t | \, | \nabla e_h^{m}| \rho ^m + C \tau _m \int _{t_{m-1}}^{t_m} \int _\varOmega | \nabla u^e | \, | \nabla e_h^{m}| \rho ^m \\&\qquad + C \frac{\tau _m}{\epsilon } \int _{t_{m-1}}^{t_m} \int _\varOmega | \nabla u^e | \, | \nabla e_h^{m}| \sqrt{\rho ^m} + C \frac{\tau _m^2}{\epsilon ^2} \int _{t_{m-1}}^{t_m} \int _{U_{\frac{3 \epsilon \pi }{4}}(t_m)} | \nabla u^e | \, | \nabla e_h^{m}| \\&\quad \le C \left[ \tau _m^{\frac{3}{2}} \left( \int _{t_{m-1}}^{t_m} \Vert u^e_t(\cdot ,t) \Vert ^2_{H^1(U_{\frac{3 \epsilon \pi }{4}}(t))} \right) ^{\frac{1}{2}}\right. \\&\qquad \left. + \frac{\tau _m^2}{\epsilon } \max _{t_{m-1} \le t \le t_m} \Vert u^e(\cdot ,t) \Vert _{H^1(U_{\frac{3 \epsilon \pi }{4}}(t))} \right] \left( \int _\varOmega | \nabla e_h^{m}|^2 \rho ^m \right) ^{\frac{1}{2}} \\&\qquad + C \frac{\tau _m^3}{\epsilon ^2} \max _{t_{m-1} \le t \le t_m} \Vert u^e(\cdot ,t) \Vert _{H^1(U_{\frac{3 \epsilon \pi }{2}}(t))} \left( \int _{U_{\frac{3 \epsilon \pi }{4}}(t_m)} | \nabla e_h^{m}|^2 \right) ^{\frac{1}{2}} \\&\quad \le \frac{\tau _m}{8} \int _\varOmega | \nabla e_h^{m}|^2 \rho ^m |\nabla \phi ^{m}|+ C \tau _m^2 \epsilon \int _{t_{m-1}}^{t_m} \bigl ( \Vert \partial _t^{\bullet } u(\cdot ,t) \Vert _{H^1(\varGamma (t))}^2 \\&\qquad + \Vert u(\cdot ,t) \Vert _{H^2(\varGamma (t))}^2 \bigr ) dt \!+\! \frac{\tau _m^2}{8} \int _\varOmega I^m_h \tilde{\rho }^m | \nabla e_h^{m}|^2 \!+\! C \tau _m^2 \epsilon \max _{t_{m-1} \le t \le t_m} \Vert u(\cdot ,t) \Vert _{H^1(\varGamma (t))}^2. \end{aligned}$$

Here we have used again that $\tau _m \le \tau \le \epsilon ^2$. In a similar way we obtain

$$\begin{aligned}&| \langle S^m_6,e_h^{m}\rangle | \le C \int _{t_{m-1}}^{t_m} \int _\varOmega \left[ | u^{e,m} - u^e| \, \rho ^m + | u^e | \, | {\varvec{v}}^m | \nabla \phi ^m | \right. \\&\left. \quad - {\varvec{v}}| \nabla \phi | | \, \rho ^m + | u^e | \, | \rho ^m - \rho | \right] | \nabla e_h^{m}| \le \frac{\tau _m}{8} \int _\varOmega | \nabla e_h^{m}|^2 \rho ^m |\nabla \phi ^{m}|\\&\quad +C \tau _m^2 \epsilon \int _{t_{m-1}}^{t_m} \left( \Vert \partial _t^{\bullet } u(\cdot ,t) \Vert _{L^2(\varGamma (t))}^2 + \Vert u(\cdot ,t) \Vert _{H^1(\varGamma (t))}^2 \right) dt \\&\quad + \frac{\tau _m^2}{8} \int _\varOmega I^m_h \tilde{\rho }^m | \nabla e_h^{m}|^2 + C \tau _m^2 \epsilon \max _{t_{m-1} \le t \le t_m} \Vert u(\cdot ,t) \Vert _{L^2(\varGamma (t))}^2 \end{aligned}$$

as well as

$$\begin{aligned} | \langle S^m_7,e_h^{m}\rangle |\le & {} C \int _{t_{m-1}}^{t_m} \int _\varOmega \left[ | f^e | \nabla \phi | - f^{e,m} | \nabla \phi ^m | \, | \, | e_h^{m}| \, \rho ^m+ | f^{e,m} | \, | e_h^{m}| \, | \rho - \rho ^m| \right] \\\le & {} C \tau _m^2 \int _\varOmega | e_h^{m}| \, \rho ^m+ C \frac{\tau _m^2}{\epsilon } \int _\varOmega | e_h^{m}| \, \sqrt{\rho ^m} + C \frac{\tau _m^3}{\epsilon ^2} \int _{U_{\frac{3 \epsilon \pi }{4}}(t_m)} | e_h^{m}| \\\le & {} \tau _m \int _\varOmega ( e_h^{m})^2 \rho ^m \!+\! C \frac{\tau _m^3}{\epsilon } + C \frac{\tau _m^3}{\epsilon ^{\frac{3}{2}}} \left( \int _\varOmega ( e_h^{m})^2 \rho ^m + \epsilon ^2 \int _{U_{\frac{3 \epsilon \pi }{4}}(t_m)} | \nabla e_h^{m}|^2 \right) ^{\frac{1}{2}} \\\le & {} C \tau _m \int _\varOmega (e_h^{m})^2 \rho ^m|\nabla \phi ^{m}|+ \frac{\tau _m^2}{8} \int _\varOmega I^m_h \tilde{\rho }^m | \nabla e_h^{m}|^2 + C \frac{\tau _m^3}{\epsilon }, \end{aligned}$$

where we have used that $| U_{\frac{3 \epsilon \pi }{4}}(t_m) | \le C \epsilon$ and again the fact that $\tau \le \epsilon ^2$. Finally, (32) and the definition of $\rho$ imply that

$$\begin{aligned} | R(\cdot ,t) | \le C \Vert u(\cdot ,t)\Vert _{W^{2,\infty }(\varGamma (t))} \text{ and } | \phi (\cdot ,t) | \le c \epsilon \text{ a.e. } \text{ on } \text{ supp } \rho (\cdot ,t), \, t \in [t_{m-1},t_m] \end{aligned}$$

so that we may estimate with the help of (27), (50) and Lemma 6

$$\begin{aligned} | \langle S^m_8,e_h^{m}\rangle |\le & {} C \int _{t_{m-1}}^{t_m} \int _\varOmega \left[ | \phi | \, | e_h^{m}| \, \rho ^m+ | \phi | \, | e_h^{m}| \, | \rho - \rho ^m| \right] \\\le & {} C \epsilon \tau _m \int _\varOmega | e_h^{m}| \, \rho ^m+ C \tau _m^2 \int _\varOmega | e_h^{m}| \, \sqrt{\rho ^m} + C \frac{\tau _m^3}{\epsilon } \int _{U_{\frac{3 \epsilon \pi }{4}}(t_m)} | e_h^{m}| \\\le & {} \tau _m \int _\varOmega ( e_h^{m})^2 \rho ^m + C \tau _m \epsilon ^3 + C \tau _m^3 \epsilon \\&\quad + C \frac{\tau _m^3}{\sqrt{\epsilon }} \left( \int _\varOmega ( e_h^{m})^2 \rho ^m + \epsilon ^2 \int _{U_{\frac{3 \epsilon \pi }{4}}(t_m)} | \nabla e_h^{m}|^2 \right) ^{\frac{1}{2}} \\\le & {} C \tau _m \int _\varOmega (e_h^{m})^2 \rho ^m|\nabla \phi ^{m}|+ \frac{\tau _m^2}{8} \int _\varOmega I^m_h \tilde{\rho }^m | \nabla e_h^{m}|^2 + C \tau _m \epsilon ^3 + C \tau _m^3 \epsilon . \end{aligned}$$

Inserting the above estimates into (51) we obtain

$$\begin{aligned}&\frac{1}{2}\int _\varOmega (e_h^{m})^2 \rho ^m|\nabla \phi ^{m}|+ \frac{\tau _m}{2} \int _\varOmega |\nabla e_h^{m}|^2 \rho ^m|\nabla \phi ^{m}|+ \frac{\tau _m^2}{4} \int _\varOmega I^m_h \tilde{\rho }^m | \nabla e_h^{m}|^2 \\&\quad \le \frac{1}{2}\int _\varOmega (e_h^{m-1})^2 \rho ^{m-1}| \nabla \phi ^{m-1}|+ C \tau _m \int _\varOmega (e_h^{m})^2 \rho ^m |\nabla \phi ^{m}|+ C \left( \frac{\tau _m^3}{\epsilon } + \tau _m \epsilon ^3 \right) \\&\quad \quad + C \tau _m \, \epsilon \left( h^2 + \frac{h^4}{\epsilon ^2} + \tau \right) \max _{t_{m-1} \le t \le t_m} \Vert u(\cdot ,t) \Vert _{H^2(\varGamma (t))}^2 \\&\quad \quad + C \epsilon \bigl ( h^4 + \tau ^2 \bigr ) \int _{t_{m-1}}^{t_m} \left( \Vert \partial ^{\bullet }_t u(\cdot ,t) \Vert _{H^2(\varGamma (t))}^2 + \Vert u(\cdot ,t) \Vert _{H^3(\varGamma (t))}^2 \right) dt. \end{aligned}$$

Choosing $\tau _2 \le \tau _1$ small enough and using (36) as well as $\tau \le \epsilon ^2$ we infer

$$\begin{aligned}&\int _\varOmega (e_h^{m})^2 \rho ^m|\nabla \phi ^{m}|+ \tau _m \int _\varOmega |\nabla e_h^{m}|^2 \rho ^m|\nabla \phi ^{m}|\\&\quad \le (1+ C \tau _m) \int _\varOmega (e_h^{m-1})^2 \rho ^{m-1}| \nabla \phi ^{m-1}|+ C \epsilon ^3 \tau _m \max _{t_{m-1} \le t \le t_m} \Vert u(\cdot ,t) \Vert _{H^2(\varGamma (t))}^2 \\&\quad + C \epsilon ^5 \int _{t_{m-1}}^{t_m} \bigl ( \Vert \partial ^{\bullet }_t u(\cdot ,t) \Vert _{H^2(\varGamma (t))}^2 + \Vert u(\cdot ,t) \Vert _{H^3(\varGamma (t))}^2 \bigr ) dt + C \tau _m \epsilon ^3. \end{aligned}$$

Summing from $m=1,\ldots ,l$, dividing by $\epsilon$ and recalling (50) we derive

$$\begin{aligned}&\frac{1}{\varepsilon }\int _\varOmega (e^l_h)^2 \rho ^l |\nabla \phi ^l| + \sum _{m=1}^l \tau _m \frac{1}{\epsilon } \int _\varOmega |\nabla e_h^{m}|^2 \rho ^m|\nabla \phi ^{m}|\\&\quad \le \frac{1}{\varepsilon }\int _\varOmega (e^0_h)^2\rho ^0 \, | \nabla \phi ^0 | + C \sum _{m=0}^{l-1} \tau _{m+1} \frac{1}{\varepsilon }\int _\varOmega (e_h^{m})^2 \rho ^m\, |\nabla \phi ^{m}|+ C \epsilon ^2. \end{aligned}$$

In order to estimate the first term on the right hand side we write $e^0_h = (I^0_h u^e_0 - u^e_0) + (u^e_0- u_h^0)$ and recall the definition (38) of $u^0_h$ as an $L^2$ projection:

$$\begin{aligned} \int _\varOmega (e^0_h)^2 \rho ^0 \, | \nabla \phi ^0 | \le C \int _{D^0_h} (e^0_h)^2 \le C \int _{D^0_h} | u^e_0 - I^0_h u^e_0 |^2 \le C \epsilon h^4 \Vert u_0 \Vert _{H^2(\varGamma (0))}^2 \end{aligned}$$

by Lemma 7. Thus

52 $$\begin{aligned}&\displaystyle \frac{1}{\varepsilon }\int _\varOmega (e^l_h)^2 \rho ^l |\nabla \phi ^l| + \sum _{m=1}^l \tau _m \frac{1}{\epsilon } \int _\varOmega |\nabla e_h^{m}|^2 \rho ^m|\nabla \phi ^{m}|\nonumber \\&\quad \le C \sum _{m=0}^{l-1} \tau _{m+1} \frac{1}{\varepsilon }\int _\varOmega (e_h^{m})^2 \rho ^m|\nabla \phi ^{m}|+ C \epsilon ^2 \end{aligned}$$

and the discrete Gronwall lemma gives

53 $$\begin{aligned} \displaystyle \max _{m=1,\ldots ,M} \frac{1}{\varepsilon }\int _\varOmega (e_h^{m})^2 \rho ^m|\nabla \phi ^{m}|\le C \epsilon ^2. \end{aligned}$$

The remainder of the proof follows from (52) and Lemma 7. $\square$

Using the result of Theorem 2 we can now also derive an error bound on the surface.

### Corollary 1

In addition to the assumptions of Theorem 2 suppose that there exists $\alpha >0$ such that $h_T \ge \alpha \epsilon$ for all $T \in {\mathcal {T}}_h$ with $| T \cap \varGamma (t) |>0, t \in [0,T]$. Then

$$\begin{aligned} \max _{m=1,\ldots ,M} \int _{\varGamma (t_m)} | u^m - u^m_h |^2 + \sum _{m=1}^M \tau _m \int _{\varGamma (t_m)} | \nabla _{\varGamma } ( u^m - u^m_h ) |^2 \le C \epsilon ^2. \end{aligned}$$

### Proof

Let us fix $m \in \lbrace 1,\ldots ,M \rbrace$ and define ${\mathcal {T}}^m_{\varGamma ,h}:= \lbrace T \in {\mathcal {T}}_h \, | \, | T \cap \varGamma (t_m) |>0 \rbrace$. Hence, given $T \in {\mathcal {T}}^m_{\varGamma ,h}$, there exists $x_T \in \varGamma (t_m)$ with $\phi ^m(x_T)=0$. We infer from (8) and (36) that for arbitrary $x \in T$

$$\begin{aligned} | \phi ^m(x) | = | \phi ^m(x) - \phi ^m(x_T) | \le c_1 | x - x_T| \le c_1 h_T \le \frac{\epsilon }{2} \cos ^2 \left( \frac{3 \pi }{8} \right) \le \frac{\epsilon \pi }{4}, \end{aligned}$$

and therefore

54 $$\begin{aligned} \rho ^m(x) \ge \frac{1}{2} \quad \text{ for } \text{ all } x \in T, \, T \in {\mathcal {T}}^m_{\varGamma ,h}. \end{aligned}$$

We now argue in a similar way as in [6], page 368. Using an interpolation inequality and an inverse estimate we infer that

$$\begin{aligned} \int _{\varGamma (t_m)} | u^m - u^m_h |^2= & {} \sum _{T \in {\mathcal {T}}^m_{\varGamma ,h}} \int _{T \cap \varGamma (t_m)} | u^m - u^m_h |^2 \\\le & {} 2 \sum _{T \in {\mathcal {T}}^m_{\varGamma ,h}} | T \cap \varGamma (t_m) | \left( \Vert d^m \Vert _{L^{\infty }(T)}^2 + \Vert e^m_h \Vert _{L^{\infty }(T)}^2 \right) \\\le & {} C \sum _{T \in {\mathcal {T}}^m_{\varGamma ,h} } | T \cap \varGamma (t_m) | \, h_T^2 \Vert \nabla u^{e,m} \Vert _{W^{1,\infty }(T)}^2 \\&+ C \sum _{T \in {\mathcal {T}}^m_{\varGamma ,h} } h_T^n h_T^{-(n+1)} \Vert e^m_h \Vert _{L^2(T)}^2 \\\le & {} C h^2 | \varGamma (t_m) | \Vert u^m \Vert _{W^{1,\infty }(\varGamma (t_m))}^2 + C \epsilon ^{-1} \sum _{T \in {\mathcal {T}}^m_{\varGamma ,h} } \int _T | e^m_h |^2 \rho ^m \, | \nabla \phi ^m |, \end{aligned}$$

where the last inequality follows from (54), (8) and the assumption that $h_T \ge \alpha \epsilon , T \in {\mathcal {T}}^m_{\varGamma ,h}$. In a similar way we obtain

$$\begin{aligned}&\int _{\varGamma (t_m)} | \nabla _{\varGamma } (u^m-u^m_h) |^2 \le C h^2 | \varGamma (t_m) | \Vert u^m \Vert _{W^{2,\infty }(\varGamma (t_m))}^2 \\&\quad + C \epsilon ^{-1} \sum _{T \in {\mathcal {T}}^m_{\varGamma ,h} } \int _T | \nabla e^m_h |^2 \rho ^m \, | \nabla \phi ^m |. \end{aligned}$$

Thus,

$$\begin{aligned}&\max _{m=1,\ldots ,M} \int _{\varGamma (t_m)} | u^m - u^m_h |^2 + \sum _{m=1}^M \tau _m \int _{\varGamma (t_m)} | \nabla _{\varGamma } ( u^m - u^m_h ) |^2 \\&\quad \le C h^2 \max _{t \in [0,T]} \Vert u(\cdot ,t) \Vert _{W^{2,\infty }(\varGamma (t))}^2 \\&\qquad + C \epsilon ^{-1} \max _{m=1,\ldots ,M} \int _{\varOmega } | e^m_h |^2 \rho ^m | \nabla \phi ^m | \!+\! C \epsilon ^{-1} \sum _{m=1}^M \tau _m \int _{\varOmega } | \nabla e^m_h |^2 \rho ^m | \nabla \phi ^m | \le C \epsilon ^2, \end{aligned}$$

by (36), (53) and (52). $$\square $$

## Numerical results

As already mentioned in Remark 2 c), the scheme (37), (38) is not fully practical. Therefore, our implementation uses the following modification: Find $u^m_h \in V^m_h,$ such that

55 $$\begin{aligned}&\int _{\varOmega } u^m_h \, v_h \, I^m_h \rho ^m \, | \nabla I^m_h \phi ^m | - \int _{\varOmega } u^{m-1}_h \, v_h \, I^{m-1}_h \rho ^{m-1} \, | \nabla I^{m-1}_h \phi ^{m-1} | \nonumber \\&\quad + \tau _m \, \int _{\varOmega } (\nabla u^m_h, \nabla v_h) \, I^m_h \rho ^m \, | \nabla I^m_h \phi ^m | - \tau _m \, \int _{\varOmega } u^m_h \, (I_h^m \hat{{\varvec{v}}}^m,\nabla v_h) \, I^m_h \rho ^m \, | \nabla I^m_h \phi ^m | \nonumber \\&\quad + \gamma \tau _m^2 \, \int _{\varOmega } I^m_h \tilde{\rho }^m ( \nabla u^m_h,\nabla v_h) = \tau _m \, \int _{\varOmega } I_h^m\hat{f}^m \, v_h \, I^m_h \rho ^m \, | \nabla I^m_h \phi ^m | \end{aligned}$$

for all $v_h \in V^m_h$ and $1 \le m \le M$. Here, $\hat{{\varvec{v}}}^m(x):= {\varvec{v}}(\hat{p}(x,t_m),t_m)$, $\hat{f}^m(x)= f(\hat{p}(x,t_m),t_m)$, where $\hat{p}(x,t)$ denotes the closest point projection of a point *x* onto $\varGamma (t)$. Setting $\hat{u}_0(x)=u_0(\hat{p}(x,0))$ we define the initial data $\hat{u}^0_h \in V^0_h$ by

56 $$\begin{aligned} \displaystyle \int _{D^0_h} \hat{u}^0_h \, v_h = \int _{D^0_h} I^0_h \hat{u}_0 \, v_h \qquad \forall v_h \in V^0_h. \end{aligned}$$

Let us remark that the evaluation of $\hat{p}(x,t)$ is easier compared to $\tilde{p}(x,t)$, which has been used to extend the data for the scheme (37), (38). However, we claim that

57 $$\begin{aligned} \tilde{p}(x,t) - \hat{p}(x,t) = O(\phi (x,t)^2). \end{aligned}$$

To see this, we first observe that $\hat{p}(x,t)$ is characterized by the conditions

$$\begin{aligned} \phi (\hat{p}(x,t),t)=0 \quad \text{ and } \quad x - \hat{p}(x,t) \perp \varGamma (t) \text{ at } \hat{p}(x,t). \end{aligned}$$

Therefore, it is not difficult to verify with the help of Taylor expansion that

$$\begin{aligned} x \!-\! \hat{p}(x,t) \!=\! \lambda (x,t) \nabla \phi (\hat{p}(x,t),t), \quad \text{ with } \lambda (x,t)\! =\! \frac{\phi (x,t)}{| \nabla \phi (\hat{p}(x,t),t)|^2}\! +\! O(\phi (x,t)^2). \end{aligned}$$

Combining this relation with (58) in the “Appendix” we find that

$$\begin{aligned} \tilde{p}(x,t) - \hat{p}(x,t)= & {} \phi (x,t) \left[ \frac{\nabla \phi (\hat{p}(x,t),t)}{| \nabla \phi (\hat{p}(x,t),t) |^2} - \frac{ \nabla \phi (x,t)}{ | \nabla \phi (x,t) |^2} \right] \\&+ O(\phi (x,t)^2) = O(\phi (x,t)^2). \end{aligned}$$

In particular, we infer from (57) that replacing $\tilde{p}$ by $\hat{p}$ in the extension of ${\varvec{v}},f$ and $u_0$ will not affect the result of Theorem 2. In contrast, it is not straightforward to handle the interpolation terms $I^m_h \rho ^m$ and $I^{m-1}_h \rho ^{m-1}$ in (55). Applying a standard interpolation estimate to $\rho ^m - I^m_h \rho ^m$ will result in a term of the form $h^2 \Vert \rho ^m \Vert _{H^2} \approx \frac{h^2}{\epsilon ^2}$, which we are currently not able to analyze. The results of our test calculations below however show that the use of the interpolation operator in (55), (56) does not lead to reduced convergence rates. More precisely we investigate the experimental order of convergence (eoc) for the following errors:

$$\begin{aligned} \mathcal {E}_1= & {} \max _{m=1,\ldots ,M} \frac{2}{\epsilon \pi } \int _{\varOmega } \, | I_h^m\hat{u}^m - u^m_h|^2 \, I^m_h \rho ^m\, | \nabla I^m_h \phi ^m |, \\ \mathcal {E}_2= & {} \frac{2}{\epsilon \pi } \sum _{m=1}^M \tau _m \int _{\varOmega } | \nabla (I_h^m \hat{u}^m- u^m_h) |^2 I^m_h \rho ^m \, | \nabla I^m_h \phi ^m |, \end{aligned}$$

where $\hat{u}^m(x)=u(\hat{p}(x,t_m),t_m)$. We use the finite element toolbox Alberta 2.0, [24], and implement a similar mesh refinement strategy to that in [2] with a fine mesh constructed in $D_h^m$ and a coarser mesh in $\varOmega \backslash D_h^m$. The linear systems appearing in each time step were solved using GMRES together with diagonal preconditioning. The values of *h* given below are such that $h:=\max _{T\in D_h^m}h_T$, $h_T=\text{ diam }(T)$.

### Remark 4

Although the analysis requires $\gamma >0$, the method works with $\gamma =0$ and produces very similar eocs to the ones displayed in the tables below for $\gamma =0.01$.

### 2D examples

We set $\varOmega = (-2.4, 2.4)^2$, $T=0.1$, and choose $\gamma =0.01, \, \epsilon =85.33 \, h$ as well as a uniform time step $\tau _m = 0.0025\varepsilon ^2, m=1,\ldots ,M$. In all our examples below $\varGamma (t)$ will be a circle $\varGamma (t)= \lbrace x \in {\mathbb {R}}^2 \, | \, | x - m(t) |= 1 \rbrace$ of radius 1 with center $m(t) \in {\mathbb {R}}^2$. In addition to ${\mathcal {E}}_1, {\mathcal {E}}_2$ we shall also investigate the errors appearing in Corollary 1. To do so we choose $L>0$ and define the following quadrature points

$$\begin{aligned} x_l(t):= m(t)+ \left( \cos \left( \frac{2 \pi l}{L}\right) , \sin \left( \frac{2 \pi l}{L}\right) \right) ^T, \quad l=0,\ldots ,L-1 \end{aligned}$$

as well as

$$\begin{aligned} \mathcal {E}_3= & {} \max _{m=1,\ldots ,M} \sum _{l=0}^{L-1}\frac{2\pi }{L}| u(x_l(t_m),t_m) - u^{m}_h(x_l(t_m)) |^2,\\ \mathcal {E}_4= & {} \sum _{m=1}^M \tau _m\sum _{l=0}^{L-1}\frac{2\pi }{L}| \nabla _{\varGamma }u(x_l(t_m),t_m) - \nabla _{\varGamma } u^{m}_h(x_l(t_m)) |^2. \end{aligned}$$

In our computations $L=200$ turned out to be sufficient.

#### Example 1

For our first example we consider the stationary unit circle $\varGamma (t)=\varGamma = S^1, t \in [0,T]$ described as the zero level set of the function $\phi (x):= x_1^2+x_2^2-1$.

The function $u(x,t):= e^{-4t} \left[ x_1 x_2 \cos (\pi t) + \frac{1}{2}(x_1^2 - x_2^2) \sin (\pi t) \right]$ is a solution of (1), (2) for the velocity field ${\varvec{v}}({x}) = \frac{\pi }{2} (x_2, -x_1)^T, f=0$ and the initial data $u_0(x)=x_1 x_2$. A similar choice of velocity appears in Example 3 in [10]. In Tables 1 and 2 we display the values of $\mathcal {E}_i$, $i=1\rightarrow 4$, together with the eocs.

**Table 1:** Errors and experimental orders of convergence for Example 1

*h*	$\varepsilon$	$\mathcal {E}_1$	$eoc_1$	$\mathcal {E}_2$	$eoc_2$
4.6875e−03	0.4	2.0565e−04	–	1.0763e−03	–
3.3146e−03	$0.2\sqrt{2}$	3.2822e−05	5.295	2.7030e−04	3.987
2.3437e−03	0.2	6.5608e−06	4.645	6.7864e−05	3.988
1.6573e−03	$0.1\sqrt{2}$	1.4513e−06	4.353	1.7017e−05	3.991
1.1719e−03	0.1	3.4022e−07	4.186	4.2668e−06	3.991

**Table 2:** Errors and experimental orders of convergence for Example 1

*h*	$\varepsilon$	$\mathcal {E}_3$	$eoc_3$	$\mathcal {E}_4$	$eoc_4$
4.6875e−03	0.4	2.7651e−05	–	4.3137e−06	–
3.3146e−03	$0.2\sqrt{2}$	8.1077e−06	3.540	1.6031e−06	2.856
2.3437e−03	0.2	2.1848e−06	3.784	5.9541e−07	2.858
1.6573e−03	$0.1\sqrt{2}$	5.6637e−07	3.895	2.3962e−07	2.626
1.1719e−03	0.1	1.4412e−07	3.949	9.6590e−08	2.622

#### Example 2

(cf. [16, Section 3.1], [26], Example 5.2) We consider the family of unit circles $\varGamma (t) = \lbrace x \in {\mathbb {R}}^2 \, | \, (x_1 + \frac{1}{2} -2t)^2 + x_2^2 =1 \rbrace$ described as the zero level set of $\phi (x,t)=(x_1 + \frac{1}{2}-2t)^2+x_2^2-1$. The function $u:S_T \rightarrow {\mathbb {R}}$, $u(x,t)= e^{-4t}(x_1 + \frac{1}{2}-2t) x_2$ is a solution of (1), (2) for the velocity field ${\varvec{v}}(x,t) = (2,0)^T,f=0$ and the initial data $u_0(x)=(x_1+\frac{1}{2})x_2$. The results are displayed in Tables 3 and 4 where we see eocs that are similar to the ones in Tables 1 and 2.

We see that the eoc for $\mathcal {E}_1$ is reducing towards 4, the eocs for $\mathcal {E}_2$ and $\mathcal {E}_3$ are close to 4 and the eoc for $\mathcal {E}_4$ is between 2 and 3 which is better than Theorem 2 predicts. Since $\mathcal {E}_1$ and $\mathcal {E}_3$ approximate $L^2$–errors, higher eocs can be expected although a corresponding proof is by no means straightforward and beyond the scope of this paper. The higher eoc for $\mathcal {E}_2$ presumably reflects a superconvergence effect because we consider $\nabla (I^m_h \hat{u}^m - u^m_h)$ rather than $\nabla ( \hat{u}^m - u^m_h)$. We expect that $\mathcal {E}_4$ will tend towards 2 if $\varepsilon ,~h$ and $\tau$ are reduced further.

**Table 3:** Errors and experimental orders of convergence for Example 2

*h*	$\varepsilon$	$\mathcal {E}_1$	$eoc_1$	$\mathcal {E}_2$	$eoc_2$
4.6875e−03	0.4	1.5537e−04	–	9.3201e−04	–
3.3146e−03	$0.2\sqrt{2}$	2.5206e−05	5.248	2.3280e−04	4.002
2.3437e−03	0.2	4.8726e−06	4.742	5.8500e−05	3.985
1.6573e−03	$0.1\sqrt{2}$	1.0558e−06	4.413	1.4776e−05	3.970
1.1719e−03	0.1	2.4507e−07	4.214	3.7865e−06	3.929

**Table 4:** Errors and experimental orders of convergence for Example 2

*h*	$\varepsilon$	$\mathcal {E}_3$	$eoc_3$	$\mathcal {E}_4$	$eoc_4$
4.6875e−03	0.4	1.8431e−05	–	3.0082e−06	–
3.3146e−03	$0.2\sqrt{2}$	5.6312e−06	3.421	1.2489e−06	2.537
2.3437e−03	0.2	1.5443e−06	3.733	4.8015e−07	2.758
1.6573e−03	$0.1\sqrt{2}$	4.0396e−07	3.869	1.9389e−07	2.616
1.1719e−03	0.1	1.0350e−07	3.929	8.1747e−08	2.492

### 3D example

#### Example 3

Here we consider the first example in Section 7 of [18] in which a family of expanding and collapsing spheres is considered such that $\varGamma (t)= \lbrace x \in {\mathbb {R}}^2 \, | \, |x|=r(t) \rbrace$ where $r(t) = 1 + \sin ^2(\pi t)$, described as the zero level set of $\phi (x,t)=x_1^2+x_2^2+x_3^2-r(t)^2$. The function $u:S_T \rightarrow {\mathbb {R}}, u(x,t)= \frac{2}{r(t)^2|x|^2} e^{-6\int _0^t\frac{1}{r^2(t)}} x_1 x_3$ is a solution of (1), (2) for the velocity field ${\varvec{v}}(x,t) = \frac{r'(t)}{|x|} x, f=0$ and the initial data $u_0(x)=\frac{2}{|x|^2}x_1 x_3$. We set $\varOmega =(-4,4)^3$, $T=0.1$ and choose $\gamma =0.01, \, \epsilon = 1.85 \, h$ as well as a uniform time step $\tau _m = 0.5h^2, m=1,\ldots ,M$. For this example we only display the errors on the surfaces which are in this case approximated by the quadrature rules

$$\begin{aligned} \mathcal {E}_3= & {} \max _{m=1,\ldots ,M} \sum _{k=0}^{2L-1} \sum _{l=0}^{L-1} \left( \frac{\pi }{L}\right) ^2 | u(x_{k,l}(t_m),t_m) - u^{m}_h(x_{k,l}(t_m)) |^2 \, \sin \left( \frac{l \pi }{L}\right) ,\\ \mathcal {E}_4= & {} \sum _{m=1}^M \tau _m \sum _{k=0}^{2L-1} \sum _{l=0}^{L-1} \left( \frac{\pi }{L}\right) ^2 | \nabla _{\varGamma }u(x_{k,l}(t_m),t_m) - \nabla _{\varGamma } u^{m}_h(x_{k,l}(t_m)) |^2 \, \sin \left( \frac{l \pi }{L}\right) , \end{aligned}$$

where

$$\begin{aligned} x_{k,l}(t)= & {} r(t) \left( \cos \left( \frac{k \pi }{L}\right) \sin \left( \frac{l \pi }{L}\right) , \sin \left( \frac{k \pi }{L}\right) \sin \left( \frac{l \pi }{L}\right) , \cos \left( \frac{l \pi }{L}\right) \right) ^T, \\&\quad k=0,\ldots ,2L-1, l=0,\ldots ,L-1. \end{aligned}$$

For the choice $L=200$ the results are displayed in Table 5, where we see eocs close to 4 for $\mathcal {E}_3$ and eocs close to 2 for $\mathcal {E}_4$.

**Fig. 1:**
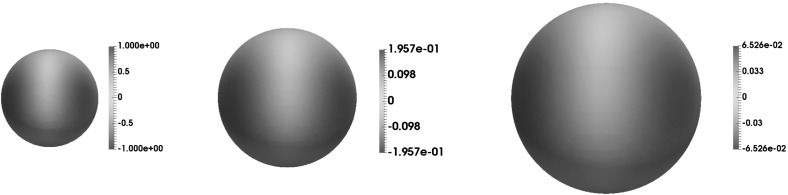
Computational results from Example 3: $u_h^m$ at times $t_m=0, 0.2,0.4$ plotted on the zero level surface of $\phi _h^m$

We conclude with Fig. 1 in which we present the approximate $u_h^m$ at times $t_m=0, 0.2,0.4$ plotted on the zero level surface of $\phi _h^m$.

**Table 5:** Errors and experimental orders of convergence for Example 3

*h*	$\varepsilon$	$\mathcal {E}_3$	$eoc_3$	$\mathcal {E}_4$	$eoc_4$
2.1651e−01	0.4	5.2016e−05	–	2.5203e−03	–
1.5309e−01	$0.2\sqrt{2}$	1.1008e−05	4.481	1.3058e−03	1.897
1.0825e−01	0.2	2.8535e−06	3.896	6.8447e−04	1.864
7.6547e−02	$0.1\sqrt{2}$	6.9422e−07	4.079	3.4543e−04	1.973

## Appendix

### Lemma 8

Suppose that *u* is a smooth solution of (1) and denote by $u^e$ the extension defined in (14). Then $u^e$ is a solution of (31) with *R* satisfying (32).

### Proof

We use the notation introduced in Sect. 2.2 and begin by deriving a formula for $\tilde{p}(x,t)$ for $x \in U_{\delta }(t), t \in [0,T]$. Define

$$\begin{aligned} \eta (\tau ) := F_t(p(x,t), (1-\tau ) \phi (x,t)), \quad \tau \in [0,1]. \end{aligned}$$

Recalling (9) and the definition of $F_t$ we have

$$\begin{aligned} \eta '(\tau ) = -\phi (x,t) \frac{ \nabla \phi ( \gamma _{p(x,t),t} ((1-\tau ) \phi (x,t)),t)}{ \left| \nabla \phi ( \gamma _{p(x,t),t} ( ( 1 - \tau ) \phi (x,t)),t) \right| ^2 }. \end{aligned}$$

Observing that $\gamma _{p(x,t),t} ( \phi (x,t) ) = F_t( p(x,t), \phi (x,t) ) = x$ and using similar arguments to calculate $\eta ''(\tau )$ we find for $k=1,\ldots ,n+1$ that

$$\begin{aligned} \eta _k'(0)= & {} - \phi (x,t) \frac{ \phi _{x_k}(x,t)}{ \left| \nabla \phi (x,t) \right| ^2}, \\ \eta _k''(0)= & {} \phi (x,t)^2 \, \sum _{l,r=1}^{n+1} \left( \delta _{kr} - \frac{2 \phi _{x_k}(x,t) \phi _{x_r}(x,t)}{ \left| \nabla \phi (x,t) \right| ^2} \right) \frac{ \phi _{x_l}(x,t) \phi _{x_l x_r}(x,t)}{\left| \nabla \phi (x,t) \right| ^4}. \end{aligned}$$

Since $\eta (1)=F_t(p(x,t),0)=\varPsi (p(x,t),t)= \tilde{p}(x,t)$, $\eta (0)=x$ we deduce with the help of Taylor’s theorem that for $k=1,\ldots ,n+1$

58 $$\begin{aligned}&\tilde{p}_k(x,t) = x_k - \phi (x,t) \frac{\phi _{x_k}(x,t)}{| \nabla \phi (x,t) |^2} + \frac{1}{2} \phi (x,t)^2 \sum _{l,r=1}^{n+1} \Bigl ( \delta _{kr}\nonumber \\&\qquad - \frac{2 \phi _{x_k}(x,t) \phi _{x_r}(x,t)}{| \nabla \phi (x,t) |^2} \Bigr ) \frac{\phi _{x_l}(x,t) \phi _{x_l x_r}(x,t)}{| \nabla \phi (x,t) |^4} + \phi (x,t)^3 r_k(x,t), \end{aligned}$$

where $r_k$ are smooth functions. Starting from (58) it is not difficult to derive formulae for $\tilde{p}_{x_i}, \tilde{p}_{x_i x_j}$ (cf. (2.9), (2.10) in [7]) and hence to deduce from (19) and (20) that

59 $$\begin{aligned}&\nabla u^e(x,t) = (I+\phi (x,t) A(x,t)) \nabla _{\varGamma }u( \tilde{p}(x,t),t) \end{aligned}$$

60 $$\begin{aligned}&\frac{1}{| \nabla \phi (x,t)|} \nabla \cdot \bigl ( | \nabla \phi (x,t)| \nabla u^e(x,t) \bigr ) = (\varDelta _{\varGamma } u)(\tilde{p}(x,t),t) \nonumber \\&\qquad +\, \phi (x,t) \left( \sum _{k,l=1}^{n+1} b_{lk}(x,t) \underline{D}_l \underline{D}_k u(\tilde{p}(x,t),t) + \sum _{k=1}^{n+1} \tilde{c}_k(x,t) \underline{D}_k u(\tilde{p}(x,t),t) \right) ,\nonumber \\ \end{aligned}$$

where $A=(a_{ik}),b_{lk}$ and $\tilde{c}_k$ are smooth. Furthermore, differentiating (58) with respect to *t* we find that

$$\begin{aligned} \tilde{p}_t(x,t)= & {} - \frac{\phi _t(x,t)}{| \nabla \phi (x,t) |^2} \nabla \phi (x,t) + \phi (x,t) q(x,t)\\= & {} V(x,t) \nu (x,t) + \phi (x,t) q(x,t), \quad q \text{ smooth } \end{aligned}$$

so that we infer from (59), (21) and (6) that

61 $$\begin{aligned}&\partial ^{\bullet }_t u^e(x,t) = u^e_t(x,t) + \bigl ( {\varvec{v}}(x,t), \nabla u^e(x,t) \bigr ) = u^e_t(x,t) \nonumber \\&\qquad + \bigl ( {\varvec{v}}(x,t), (I+\phi (x,t) A(x,t)) \nabla _{\varGamma }u( \tilde{p}(x,t),t) \bigr )\nonumber \\&\quad = \partial ^{\bullet }_t u (\tilde{p}(x,t),t) + \bigl ( ({\varvec{v}}(x,t) - {\varvec{v}}(\tilde{p}(x,t),t)), \nabla _{\varGamma } u(\tilde{p}(x,t),t) \bigr ) \nonumber \\&\qquad + \,V(x,t) \bigl ( (\nu (x,t) - \nu (\tilde{p}(x,t),t)), \nabla _{\varGamma } u(\tilde{p}(x,t),t) \bigr )\nonumber \\&\qquad +\, \phi (x,t) \bigl ( q(x,t) + A(x,t)^T {\varvec{v}}(x,t), \nabla _{\varGamma } u(\tilde{p}(x,t),t) \bigr ). \end{aligned}$$

The fundamental theorem of calculus together with (58) implies that

$$\begin{aligned} {\varvec{v}}(x,t) - {\varvec{v}}(\tilde{p}(x,t),t)= & {} \int _0^1 D {\varvec{v}}(sx +(1-s) \tilde{p}(x,t),t) ds \, (x - \tilde{p}(x,t)) \\= & {} \phi (x,t) \, \tilde{q}(x,t) \end{aligned}$$

for some smooth $\tilde{q}$. Arguing in the same way for the corresponding difference involving $\nu$ we infer from (61)

62 $$\begin{aligned} \displaystyle \partial ^{\bullet }_t u^e(x,t) = \partial ^{\bullet }_t u (\tilde{p}(x,t),t) + \phi (x,t) \sum _{k=1}^{n+1} \hat{c}_k(x,t) \underline{D}_k u(\tilde{p}(x,t),t), \end{aligned}$$

where $\hat{c}_k$ are smooth. Finally, since $\nabla _{\phi } \cdot {\varvec{v}}(\tilde{p}(x,t),t) = \nabla _{\varGamma } \cdot {\varvec{v}}(\tilde{p}(x,t),t)$ we have

63 $$\begin{aligned} u^e(x,t) \nabla _{\phi } \cdot {\varvec{v}}(x,t)= & {} u(\tilde{p}(x,t),t) \nabla _{\phi } \cdot {\varvec{v}}(\tilde{p}(x,t),t)\nonumber \\&+ u(\tilde{p}(x,t),t) \bigl ( \nabla _{\phi } \cdot {\varvec{v}}(x,t) - \nabla _{\phi } \cdot {\varvec{v}}(\tilde{p}(x,t),t) \bigr ) \nonumber \\= & {} u(\tilde{p}(x,t),t) \nabla _{\varGamma } \cdot {\varvec{v}}(\tilde{p}(x,t),t) + u(\tilde{p}(x,t),t) \phi (x,t) \bar{r}(x,t). \nonumber \\ \end{aligned}$$

Here, the second term has been rewritten in a similar way as above for some smooth $\bar{r}$. Combining (60)–(63) we deduce that the extension $u^e$ of a function *u* solving (1) satisfies (31), where *R* has the form (32). $\square$
